# Single nucleotide polymorphism in the 3′ untranslated region of *LPP* is a risk factor for lung cancer: a case-control study

**DOI:** 10.1186/s12885-018-5241-5

**Published:** 2019-01-08

**Authors:** Shouchun Yan, Rong Sun, Shan Wu, Tianbo Jin, Shanshan Zhang, Fanglin Niu, Jingjie Li, Mingwei Chen

**Affiliations:** 1 Department of Respiratory Medicine, The First Affiliated Hospital of School of Medicine of Xi’an Jiaotong University, Xi’an, 710061 Shaanxi China; 2 Department of Emergency Medicine, Xi’an No.1 hospital, Xi’an, 710002 Shaanxi China; 3 Department of Emergency Medicine, Xi’an GaoXin Hospital, Xi’an, 710075 Shaanxi China; 4 Department of Respiratory Medicine, Xi’an No.1 hospital (Gaoling District), Xi’an, 710299 Shaanxi China; 5 Key Laboratory of Resource Biology and Biotechnology in Western China (Northwest University), Ministry of Education, Xi’an, 710069 Shaanxi China

**Keywords:** Lung cancer, *LPP*, Polymorphism, Genetic susceptibility

## Abstract

**Background:**

Single nucleotide polymorphisms (SNPs) in 3′-untranslated region (UTR) of genes related with cell-matrix adhesions and migration might affect miRNA binding and potentially affect the risk of cancer. The present study aimed to screen SNPs in 3′ UTR of cancer-related genes and investigate their contribution to the susceptibility of lung cancer.

**Methods:**

Seven SNPs were selected and genotyped in a case-control study (322 lung cancer patients and 384 controls) among Chinese Han population. Odds ratio (OR) and 95% confidence intervals (CIs) were calculated by logistic regression adjusted for age and gender in multiple genetic models.

**Results:**

In stratified analyses by gender, three (rs1064607, rs3796283 and rs2378456) of *LPP* gene were associated with a significantly increased susceptibility for lung cancer among male population. Besides, *LPP* rs2378456 weakened lung cancer risk in female. *LPP* rs1064607 polymorphism was significantly correlated with increased risk of lung adenocarcinoma. Furthermore, AA genotype of *TNS3* rs9876 polymorphism was associated with lymphatic metastasis.

**Conclusion:**

Our results provides evidence for the impact of *LPP* polymorphisms on the susceptibility to lung cancer in Chinese population.

**Electronic supplementary material:**

The online version of this article (10.1186/s12885-018-5241-5) contains supplementary material, which is available to authorized users.

## Background

Lung cancer is the most frequently diagnosed cancers and the leading causes of cancer death in men, both overall and in less-developed countries. According to Global Cancer Statistics 2018, approximately 2.1 million new lung cancer cases were diagnosed, accounting for 11.6% of total cancer diagnoses made last year [1]. Lung cancer is also the most prevalent cancer and the main leading cause of cancer death in Chinese with significantly high age-standardized incidence and mortality rates [2]. Despite significant treatment breakthroughs, the five-year survival rate for patients with lung cancer is less than 10%, and most lung patients are diagnosed at an advanced stage [3]. Tobacco exposure is the commonly accepted primary cause of lung cancer, while and other occupational and environmental factors, such as asbestos, heavy metal, radiation and air pollution also contribute to its incidence. Additionally, individual variation, including age, sex, ethnicity and body weight and genetic susceptibility exert an important influence on the etiology of lung cancer [4]. Genetic polymorphisms are now recognized as a major cause of the disease, and the causal association between lung cancer and genetic polymorphisms has been proven in many experimental and epidemiological studies [5–7].

Cell migration plays a critical role in many biological processes and is implicated in cancer invasion and metastasis [8]. Specifically, *LPP* (lipoma preferred partner/LIM domain containing preferred translocation partner in lipoma), *TNS3* (Tensin 3) and *NR5A2* (nuclear receptor subfamily 5 group A member 2) encode proteins important in cell adhesion and migration. Recent data have shown that LPP participates in the formation of disorganized micro-vessels within tumor tissue, leading to increased migration and invasion following epithelial-mesenchymal transition [9, 10]. It has previously been shown that TNS3 is deregulated in cancer and has implications in cell migration, invasion and tumorigenesis [11]. NR5A2, also known as LRH-1 (liver receptor homolog-1) is essential for diverse biological activities, including cell proliferation and differentiation, embryonic development, and cholesterol metabolism [12]. Expression dysregulation and certain variants of these genes are associated with the risk of various cancers, including lung cancer [13–15].

Variations in the 3′-untranslated region (3′-UTRs) of genes may affect their expression by reinforcing, weakening, or disrupting miRNA-mRNA interactions [16]. Many studies of 3’UTR single nucleotide polymorphisms (SNPs) and lung cancer have provided insight into lung carcinogenesis, development of diagnostic and prognostic markers, and discovery of novel therapeutic approaches [17]. However, we did not find previous reports on the association between the 3′UTRs of *LPP*, *TNS3*, and *NR5A2* and lung cancer risk. Thus, we sought to identify relevant 3′UTR SNPs within these genes and to assess their effects on lung cancer susceptibility in the Han Chinese population using a case-control study approach.

## Materials and methods

### Study subjects

Our case-control study included 322 patients with lung cancer and 384 cancer-free control subjects. All participants were genetically unrelated Han Chinese. Lung cancer patients were enrolled in the First Affiliated Hospital of Xi′an Jiaotong University (Xi′an, China). The inclusion criteria for patients were patients with primary lung cancer who was newly diagnosed and histologically confirmed according to International Classification of Diseases for Oncology, no familial history and no cancer history. The exclusion criteria of the patient group were patients with any other malignancy and pulmonary diseases, such as chronic obstructive pulmonary disease, pneumothorax, and asthma. No age, gender, tumor histology, or stage restrictions were applied; however, patients with prior cancer history were excluded from this study. No lung cancer patients had received either radiotherapy or chemotherapy before blood sampling. Healthy controls without history of any cancer were randomly recruited from the physical examination center of the same hospital during the similar period, when they had visited for an annual health examination. The exclusion criteria for the control group were any lung cancer family history of more than three generations, tuberculosis and chronic respiratory disease. At the time of recruitment, each subject was personally interviewed by trained personnel using a structured questionnaire to obtain information on the demographic information.

All participants were voluntary recruited and provided written informed consent before taking part in this research. This study was approved by the Research Ethics Committee of the First Affiliated Hospital of Xi′an Jiaotong University, and in compliance with the Declaration of Helsinki. The design and performance of this study involving human subjects were obviously described in a research protocol.

### SNP selection

We selected three cancer-related genes (*NR5A2*, *LPP* and *TNS3* gene) to explore the relationship between their 3’-UTR SNPs and lung cancer risk in our study population. The 3′UTR sequences of these candidate genes were identified using NCBI database of nucleotide (https://www.ncbi.nlm.nih.gov/nuccore/). The online software of miRbase (http://www.mirbase.org/), miRDB (http://www.mirdb.org) and TargetScan 7.1 (http://www.targetscan.org) were used to choose SNPs in putative miRNA binding sites within 3′UTR of each gene. Information regarding genetic variations of these 3′UTR SNP was obtained by an extensive search of the dbSNP database (http://www.ncbi.nlm.nih.gov/projects/SNP/), and UCSC genome browser (http://genome.ucsc.edu/). SNPs with a minor allele frequency (MAF) greater than 5% in Chinese Han population were selected based on the HapMap Han Chinese in Beijing (CHB) database. Seven SNPs (rs2246209 and rs1056426 in *NR5A2*; rs1064607, rs3796283 and rs2378456 in *LPP*; rs3750163 and rs9876 in *TNS3*) were ultimately chosen as the candidate SNPs for the further evaluation. Using these bioinformatics tools, two miRNAs (hsa-miR-144-3p, and hsa-miR-182-5p) were identified that potentially bind to a stretch of sequence harboring candidate SNPs in the 3′ UTR of *NR5A2*, *LPP* and *TNS3*.

### SNP genotyping

Peripheral blood (5 mL) from each participant was collected and stored in Vacutainer tubes (BD Franklin Lakes, NJ) containing anticoagulant of EDTA. Genomic DNA was isolated using the QIAGEN DNA Extraction Kit (QIAGEN, Valencia, CA, USA), following the manufacturer′s protocol. Purity and concentration of the DNA samples were determined by a NanoDrop2000c Spectrophotometer (ThermoFisher Scientific, Wilmington, DE, EUA). All DNA samples were suspended in TE buffer and stored at − 80 °C for later analysis.

Genotypes of SNPs were detected using the Agena MassARRAY system (Agena, San Diego, CA, U.S.A.) by two laboratory personnel independently in a double-blinded fashion. Primers for amplification and single base extension were designed with Agena MassARRAY Assay Design 3.0 Software. Sequence data was collected and analyzed by Agena Typer 4.0 Software. Meanwhile, approximately 10% of samples were randomly selected to repeat genotyping, and the reproducibility was 100%.

### Statistical analysis

Independent sample Student′s t test (for continuous variables) and U Mann Whitney test (for categorical variables) were used to evaluate the differences in the distribution of demographic characteristics between cases and controls. Hardy-Weinberg equilibrium (HWE) was assessed using the Chi-square test to compare the observed and expected genotype frequencies in controls. The distribution of SNPs allele and genotype frequencies of cases and controls were compared with a Pearson Chi-squared test or Fisher′s exact test. Associations between genotype and lung cancer risk were evaluated by calculating odds ratios (ORs) and 95% confidence intervals (CIs) using logistic regression model with and without adjustment for age and gender. The wild-type allele was used as a reference. Multiple inheritance models (genotype, dominant, recessive and log-additive) was estimated using logistic regression analysis with adjustments for the potential lung risk factors (age and gender) by SNPstats online tools software (http://bioinfo.iconcologia.net/snpstats/start.htm). The analyses of joint effects were further stratified by age (≤ 50 and >  50 years) and gender (male and female). Power and Sample Size (PS) Calculation software (http://biostat.mc.vanderbilt.edu/wiki/Main/PowerSampleSize) was used to calculate the power of the signifcant difference. Haplotypes were reconstructed with Haploview software package (version 4.2) and SNPstats software. In haplotype analysis, haplotype frequencies less than 0.01 were omitted. Besides, multifactor dimensionality reduction (MDR) (version 3.0.2) was employed to identify the potential interactions of these SNPs in *LPP* on the risk of lung cancer. All the statistical analyses were performed with SPSS v17.0 (IBM Analytics, Chicago, IL, USA), and two-sided *p* value < 0.05 indicated statistical significance.

## Results

Our study included 706 subjects including 322 patients with lung cancer (245 males and 77 females) and 384 healthy controls (278 males and 106 females). There were no statistically significant differences (*p* = 0.265) on the gender distribution between the case and control groups. The average age among cases and controls was 59.00 ± 9.83 years and 51.16 ± 11.50 years, respectively. However, the result revealed the age distribution was statistically significant differences (*p* < 0.001). A summary of the participants′ demographic characteristics was summarized in Table 1. Lung cancer patients were consisted of 150 adenocarcinomas, 98 squamous cell carcinomas, and 74 small cell adenocarcinomas.

**Table 1:** Characteristics of patients with lung cancer and controls

Characteristics	Patients (*n* = 322)	Controls (*n* = 384)
Age, years	59.00 ± 9.83	51.16 ± 11.50
≤ 50 years	58 (18.01%)	156 (40.62%)
> 50 years	264 (91.99%)	228 (59.38%)
Gender		
Male	245 (76.1%)	278 (72.4%)
Female	77 (23.9%)	106 (27.6%)
Histology		
Adenocarcinoma	150	
Squamous cell	98	
small cell carcinoma	74	
Stage		
I-II	95	
III-IV	213	
Missing	34	
Lymphatic metastasis		
Yes	193	
No	127	

Details of selected SNPs were described in Table 2. These 3′UTR SNPs were successfully genotyped for further analysis, and the call rate was above 99%. MAF of all SNPs was greater than 5% and the observed genotype frequencies of all SNPs in the control groups were in HWE (*p* > 0.05).

**Table 2:** Basic Information about the candidate SNPs in this study

Gene	SNP ID	Chr: Position	Role	Alleles	Nucleotide change	MAF		O(HET)	E(HET)	*p*-value for HWE	Call rate (%)
				(minor/major)		Cases	Controls				
NR5A2	rs2246209	1:200145533	UTR-3	A/G	2195G > A	0.301	0.313	0.417	0.430	0.553	100%
NR5A2	rs1056426	1:200146403	UTR-3	C/T	3065 T > C	0.225	0.238	0.367	0.363	0.889	99.72%
LPP	rs1064607	3:188595672	UTR-3	C/G	3405G > C	0.405	0.365	0.453	0.463	0.661	99.86%
LPP	rs3796283	3:188602952	UTR-3	G/A	10684A > G	0.447	0.414	0.474	0.485	0.674	99.86%
LPP	rs2378456	3:188603007	UTR-3	C/G	10739C > G,	0.473	0.453	0.483	0.496	0.608	99.01%
TNS3	rs3750163	7:47317510	UTR-3	A/G	164G > A	0.087	0.079	0.148	0.146	1.000	100%
TNS3	rs9876	7:47315291	UTR-3	A/G	2383G > A	0.475	0.487	0.464	0.500	0.154	99.86%

*SNP* single nucleotide polymorphism, *MAF* minor allele frequency, *O(HET)* observed heterozygosity, *E(HET)* expected heterozygosity, *HWE* Hardy-Weinberg equilibrium. *p* values were calculated with Pearson′s χ^2^ tests; *p* < 0.05 indicates statistical significance

Differences in SNPs genotype and allele frequencies between cases and controls were compared by Chi-squared test and odds ratios (ORs) to evaluate the associations with the risk of lung cancer, as showed in Table 3. The minor allele of each SNP as a risk factor was compared to the wild-type (major) allele. However, we had not found that any SNPs were significantly different of genotypes and allele frequencies between lung cancer cases and healthy population (Table 3). Multiple inheritance models (dominant, recessive, and additive models) were applied to analyze potential association by logistic regression analysis adjusted for age and gender. Again, we observed no statistically significant differences between patients and controls (*p* > 0.05, Additional file 1: Table S1).

**Table 3:** The genotype and allele frequencies of seven 3′UTR polymorphisms in lung cancer patients and controls

Gene	SNP ID	Genotype							Allele				
					Adjusted analysis		Crude analysis						
			Control	Case	OR (95% CI)	*p*	OR (95% CI)	*p*		Control	Case	OR (95% CI)	*p*
NR5A2	rs1056426	TT	222 (57.8%)	197 (61.6%)	1.00	0.270	1.00		T	585 (76.2%)	496 (77.5%)	1.00	0.560
		CT	141 (36.7%)	102 (31.9%)	0.78 (0.55–1.09)		0.82 (0.59–1.12)	0.380	C	183 (23.8%)	144 (22.5%)	0.93(0.72–1.19)	
		CC	21 (5.5%)	21 (6.6%)	1.15 (0.59–2.24)		1.13 (0.60–2.13)						
NR5A2	rs2246209	GG	184 (47.9%)	160 (49.7%)	1.00	0.940	1.00		G	528 (68.8%)	450 (69.9%)	1.00	0.650
		GA	160 (41.7%)	130 (40.4%)	0.98 (0.70–1.37)		0.93 (0.68–1.28)	0.890	A	240 (31.3%)	194 (30.1%)	0.95(0.76–1.19)	
		AA	40 (10.4%)	32 (9.9%)	0.91 (0.53–1.56)		0.92 (0.55–1.53)						
LPP	rs1064607	GG	157 (40.9%)	111 (34.6%)	1.00	0.280	1.00		G	488 (63.5%)	382 (59.5%)	1.00	0.120
		GC	174 (45.3%)	160 (49.8%)	1.25 (0.89–1.77)		1.30 (0.94–1.80)	0.230	C	280 (36.5%)	260 (40.5%)	1.19(0.96–1.47)	
		CC	53 (13.8%)	50 (15.6%)	1.41 (0.87–2.30)		1.33 (0.85–2.11)						
LPP	rs3796283	AA	134 (34.9%)	94 (29.3%)	1.00	0.490	1.00		A	450 (58.6%)	355 (55.3%)	1.00	0.210
		GA	182 (47.4%)	167 (52%)	1.24 (0.86–1.77)		1.31 (0.93–1.83)	0.280	G	318 (41.4%)	287 (44.7%)	1.14(0.93–1.41)	
		GG	68 (17.7%)	60 (18.7%)	1.20 (0.76–1.92)		1.26 (0.81–1.95)						
LPP	rs2378456	GG	117 (30.6%)	88 (27.9%)	1.00	0.890	1.00		G	419 (54.7%)	333 (52.7%)	1.00	0.450
		GC	185 (48.3%)	157 (49.7%)	1.08 (0.74–1.56)		1.13 (0.80–1.60)	0.730	C	347 (45.3%)	299 (47.3%)	1.08(0.88–1.34)	
		CC	81 (21.1%)	71 (22.5%)	1.11 (0.71–1.74)		1.17 (0.76–1.78)						
TNS3	rs3750163	GG	325 (84.6%)	270 (83.8%)	1.00	0.370	1.00		G	707 (92.1%)	588 (91.3%)	1.00	0.610
		GA	57 (14.8%)	48 (14.9%)	0.93 (0.60–1.44)		1.01 (0.67–1.54)	0.580	A	61 (7.9%)	56 (8.7%)	1.10(0.76–1.61)	
		AA	2 (0.5%)	4 (1.2%)	3.25 (0.56–18.93)		2.41 (0.44–13.24)						
TNS3	rs9876	GG	108 (28.1%)	86 (26.8%)	1.00	0.430	1.00		G	394 (51.3%)	337 (52.5%)	1.00	0.660
		AG	178 (46.4%)	165 (51.4%)	1.15 (0.79–1.67)		1.16 (0.82–1.66)	0.360	A	374 (48.7%)	305 (47.5%)	0.95(0.77–1.18)	
		AA	98 (25.5%)	70 (21.8%)	0.89 (0.57–1.38)		0.90 (0.59–1.36)						

*OR* odds ratio, *95% CI* 95% confidence interval

*p* < 0.05 indicates statistical significance

We next performed a stratified analysis according to age and gender to evaluate the effect of these 3′UTR SNPs on lung cancer risk. Stratified analysis by gender revealed significant associations between three SNPs and the risk of lung cancer, as displayed in Table 4 and Additional file 1: Table S2. These three polymorphisms (rs1064607, rs3796283 and rs2378456) in *LPP,* which increased the risk of lung cancer among males, were identified using the dominant model (rs1064607, GC-CC vs GG, OR = 1.57, CI = 1.06–2.33, *p* = 0.024; rs3796283, GA-GG vs AA, OR = 1.67, CI = 1.10–2.51, *p* = 0.014; and rs2378456, GC-CC vs GG, OR = 1.55, CI = 1.02–2.38, *p* = 0.041), with power values of 0.799, 0.866, and 0.718, respectively. We also determined that the genotype ″GA″ of rs3796283 increased the risk of lung cancer in males under genotype model (OR = 1.76, CI = 1.14–2.71, *p* = 0.036, power = 0.955). Conversely, rs2378456 polymorphism in *LPP* was associated with reduced susceptibility of lung cancer in females under genotype (GC vs GG, OR = 0.37, 95% CI = 0.18–0.76, *p* = 0.022, power = 1.000) and dominant (GC-CC vs GG, OR = 0.44, 95% CI = 0.23–0.84, *p* = 0.012, power = 0.998) model. Interaction analysis did not reveal any significant associations between these SNPs and lung cancer risk with respect to age (Additional file 1: Table S3).

**Table 4:** The relationship of seven 3′UTR polymorphisms with lung cancer according to the stratification by gender adjusted by gender + age

SNP ID	Model	Genotype	Male						Female					
			Control	Case	Adjusted analysis		Crude analysis		Control	Case	Adjusted analysis		Crude analysis	
					OR (95%CI)	*p*	OR (95%CI)	*p*			OR (95%CI)	*p*	OR (95%CI)	*p*
rs1064607	Allele	G	358 (64.4%)	286 (58.6%)	1.00	0.055			130 (61.3%)	96 (62.3%)	1.00	0.840		
		C	198 (35.6%)	202 (41.4%)	1.28 (0.99–1.64)				82 (38.7%)	58 (37.7%)	0.96 (0.62–1.47)			
	Codominant	G/G	118 (42.5%)	78 (32%)	1.00	0.078	1.00		39 (36.8%)	33 (42.9%)	1.00	0.370	1.00	
		G/C	122 (43.9%)	130 (53.3%)	**1.58 (1.04–2.39)**		**1.61 (1.10–2.35)**	**0.043**	52 (49.1%)	30 (39%)	0.68 (0.35–1.30)		0.68 (0.36–1.30)	0.390
		C/C	38 (13.7%)	36 (14.8%)	1.54 (0.85–2.80)		1.43 (0.84–2.45)		15 (14.2%)	14 (18.2%)	1.12 (0.47–2.68)		1.10 (0.47–2.62)	
	Dominant	G/G	118 (42.5%)	78 (32%)	1.00	**0.024**	1.00	**0.013**	39 (36.8%)	33 (42.9%)	1.00	0.410	1.00	0.410
		G/C-C/C	160 (57.5%)	166 (68%)	**1.57 (1.06–2.33)**		**1.57 (1.10–2.25)**		67 (63.2%)	44 (57.1%)	0.77 (0.42–1.42)		0.78 (0.43–1.41)	
	Recessive	G/G-G/C	240 (86.3%)	208 (85.2%)	1.00	0.530	1.00	0.720	91 (85.8%)	63 (81.8%)	1.00	0.440	1.00	0.460
		C/C	38 (13.7%)	36 (14.8%)	1.19 (0.69–2.05)		1.09 (0.67–1.79)		15 (14.2%)	14 (18.2%)	1.38 (0.62–3.08)		1.35 (0.61–2.99)	
	Log-additive	–	–	–	1.32 (1.00–1.74)	0.053	1.29 (1.00–1.66)	0.052	–	–	0.96 (0.63–1.47)	0.870	0.96 (0.63–1.46)	0.850
rs3796283	Allele	A	332 (59.7%)	263 (53.9%)	1.00	0.058			118 (55.7%)	92 (59.7%)	1.00	0.440		
		G	224 (40.3%)	225 (46.1%)	1.27 (0.99–1.62)				94 (44.3%)	62 (40.3%)	0.85 (0.56–1.29)			
	Codominant	A/A	104 (37.4%)	64 (26.2%)	1.00	**0.036**	1.00		30 (28.3%)	30 (39%)	1.00	0.160	1.00	
		G/A	124 (44.6%)	135 (55.3%)	**1.76 (1.14–2.71)**		**1.77 (1.19–2.63)**	**0.017**	58 (54.7%)	32 (41.6%)	0.52 (0.27–1.03)		0.55 (0.28–1.07)	0.190
		G/G	50 (18%)	45 (18.4%)	1.43 (0.81–2.50)		1.46 (0.88–2.43)		18 (17%)	15 (19.5%)	0.80 (0.34–1.89)		0.83 (0.36–1.95)	
	Dominant	A/A	104 (37.4%)	64 (26.2%)	1.00	**0.014**	1.00	**0.006**	30 (28.3%)	30 (39%)	1.00	0.100	1.00	0.130
		G/A-G/G	174 (62.6%)	180 (73.8%)	**1.67 (1.10–2.51)**		**1.68 (1.16–2.45)**		76 (71.7%)	47 (61%)	0.59 (0.31–1.11)		0.62 (0.33–1.15)	
	Recessive	A/A-G/A	228 (82%)	199 (81.6%)	1.00	0.980	1.00	0.890	88 (83%)	62 (80.5%)	1.00	0.680	1.00	0.660
		G/G	50 (18%)	45 (18.4%)	1.01 (0.61–1.65)		1.03 (0.66–1.61)		18 (17%)	15 (19.5%)	1.17 (0.55–2.52)		1.18 (0.55–2.53)	
	Log-additive	–	–	–	1.26 (0.96–1.66)	0.095	1.27 (0.99–1.63)	0.056	–	–	0.83 (0.54–1.27)	0.380	0.84 (0.55–1.29)	0.430
rs2378456	Allele	G	312 (56.3%)	245 (50.8%)	1.00	0.077			107 (50.5%)	88 (58.7%)	1.00	0.120		
		C	242 (43.7%)	237 (49.2%)	1.25 (0.98–1.59)				105 (49.5%)	62 (41.3%)	0.72 (0.47–1.09)			
	Codominant	G/G	93 (33.6%)	58 (24.1%)	1.00	0.100	1.00		24 (22.6%)	30 (40%)	1.00	**0.022**	1.00	
		G/C	126 (45.5%)	129 (53.5%)	**1.63 (1.04–2.56)**		**1.64 (1.09–2.47)**	0.053	59 (55.7%)	28 (37.3%)	**0.37 (0.18–0.76)**		**0.38 (0.19–0.76)**	**0.023**
		C/C	58 (20.9%)	54 (22.4%)	1.39 (0.80–2.40)		1.49 (0.91–2.45)		23 (21.7%)	17 (22.7%)	0.60 (0.26–1.38)		0.59 (0.26–1.35)	
	Dominant	G/G	93 (33.6%)	58 (24.1%)	1.00	**0.041**	1.00	**0.017**	24 (22.6%)	30 (40%)	1.00	**0.012**	1.00	**0.012**
		G/C-C/C	184 (66.4%)	183 (75.9%)	**1.55 (1.02–2.38)**		**1.59 (1.08–2.35)**		82 (77.4%)	45 (60%)	**0.44 (0.23–0.84)**		**0.44 (0.23–0.84)**	
	Recessive	G/G-G/C	219 (79.1%)	187 (77.6%)	1.00	0.940	1.00	0.690	83 (78.3%)	58 (77.3%)	1.00	0.830	1.00	0.880
		C/C	58 (20.9%)	54 (22.4%)	1.02 (0.64–1.62)		1.09 (0.72–1.66)		23 (21.7%)	17 (22.7%)	1.08 (0.53–2.22)		1.06 (0.52–2.15)	
	Log-additive	–	–	–	1.21 (0.92–1.58)	0.180	1.25 (0.97–1.59)	0.078	–	–	0.73 (0.48–1.11)	0.140	0.72 (0.48–1.10)	0.130

*OR* odds ratio, *95% CI* 95% confidence interval

Bold indicates statistical signifcance (*p* < 0.05)

We further assessed the association between these SNPs and clinic-pathological features, including histological subgroups, clinical stage (I-II vs. III-IV) and lymphatic metastatic stats (non-metastasis vs. metastasis). The results indicated that *LPP* rs1064607 polymorphism was significantly correlated with increased risk of lung adenocarcinoma (allele model, OR = 1.35, 95% CI: 1.03–1.77, *p* = 0.030; and additive model, OR = 1.38, 95% CI: 1.04–1.83, *p* = 0.026, Table 5). Moreover, AA genotype of *TNS3* rs9876 polymorphism had a significantly lower risk of lymphatic metastasis (AA vs. GG-AG, OR = 0.52, 95% CI: 0.30–0.89, *p* = 0.017, Table 6). Nevertheless, we failed to observe significant differences between clinic-pathological features and other polymorphisms (Additional file 1: Table S4, S5, S6, S7, S8).

**Table 5:** The relationship of seven 3′UTR polymorphisms with lung cancer according to the histology stratification

SNP ID	Model	Genotype	Control	Adenocarcinoma			Squamous cell carcinoma			Small cell carcinoma		
				Case	OR (95% CI)	*p*–value	Case	OR (95% CI)	*p*–value	Case	OR (95% CI)	*p*–value
rs1064607	Allele	G	488 (63.5%)	169 (56.3%)	1.00	**0.030**	121 (62.7%)	1.00	0.762	92 (62.2%)	1.00	0.750
		C	280 (36.5%)	131 (43.7%)	**1.35 (1.03–1.77)**		73 (37.6%)	1.05 (0.76–1.46)		56 (37.8%)	1.06 (0.74–1.53)	
	Codominant	G/G	157 (40.9%)	47 (31.3%)	1.00	0.085	37 (38.1%)	1.00	0.760	27 (36.5%)	1.00	0.780
		G/C	174 (45.3%)	75 (50%)	1.39 (0.89–2.16)		47 (48.5%)	1.22 (0.72–2.07)		38 (51.4%)	1.20 (0.69–2.10)	
		C/C	53 (13.8%)	28 (18.7%)	1.90 (1.06–3.42)		13 (13.4%)	1.13 (0.52–2.47)		9 (12.2%)	0.99 (0.43–2.31)	
	Dominant	G/G	157 (40.9%)	47 (31.3%)	1.00	0.053	37 (38.1%)	1.00	0.480	27 (36.5%)	1.00	0.590
		G/C-C/C	227 (59.1%)	103 (68.7%)	1.50 (0.99–2.28)		60 (61.9%)	1.20 (0.73–1.98)		47 (63.5%)	1.16 (0.68–1.97)	
	Recessive	G/G-G/C	331 (86.2%)	122 (81.3%)	1.00	0.096	84 (86.6%)	1.00	0.970	65 (87.8%)	1.00	0.780
		C/C	53 (13.8%)	28 (18.7%)	1.57 (0.93–2.66)		13 (13.4%)	1.02 (0.49–2.10)		9 (12.2%)	0.89 (0.41–1.95)	
	Log-additive	–	–	–	**1.38 (1.04–1.83)**	**0.026**	–	1.10 (0.77–1.58)	0.590	–	1.05 (0.72–1.53)	0.810

*SNP* single nucleotide polymorphism, *OR* odds ratio, *95% CI* 95% confidence interval. *p* values were adjusted by gender and age

Bold indicates statistical signifcance (*p* < 0.05)

**Table 6:** Relationship of lymphatic metastatic status with 3′UTR polymorphisms in lung cancer patients adjusted by gender and age

SNP ID	Model	Genotype	Non-Metastasis	Metastasis	Adjusted analysis		Crude analysis	
					OR (95%CI)	*p*	OR (95%CI)	*p*
rs9876	Allele	G	121 (48.0%)	216 (55.4%)	1.00	0.068		
		A	131 (52.0%)	174 (44.6%)	0.74 (0.54–1.02)			
	Codominant	G/G	31 (24.6%)	55 (28.2%)	1.00	0.059	1.00	
		A/G	59 (46.8%)	106 (54.4%)	1.01 (0.59–1.74)		1.01 (0.59–1.74)	0.065
		A/A	36 (28.6%)	34 (17.4%)	0.52 (0.28–1.00)		0.53 (0.28–1.01)	
	Dominant	G/G	31 (24.6%)	55 (28.2%)	1.00	0.460	1.00	0.480
		A/G-A/A	95 (75.4%)	140 (71.8%)	0.83 (0.49–1.38)		0.83 (0.50–1.39)	
	Recessive	G/G-A/G	90 (71.4%)	161 (82.6%)	1.00	**0.017**	1.00	**0.019**
		A/A	36 (28.6%)	34 (17.4%)	**0.52 (0.30–0.89)**		**0.53 (0.31–0.90)**	
	Log-additive	–	–	–	0.73 (0.53–1.01)	0.058	0.74 (0.53–1.02)	0.063

*OR* odds ratio, *95% CI* 95% confidence interval

Bold indicates statistical signifcance (*p* < 0.05)

**Fig. 1:**
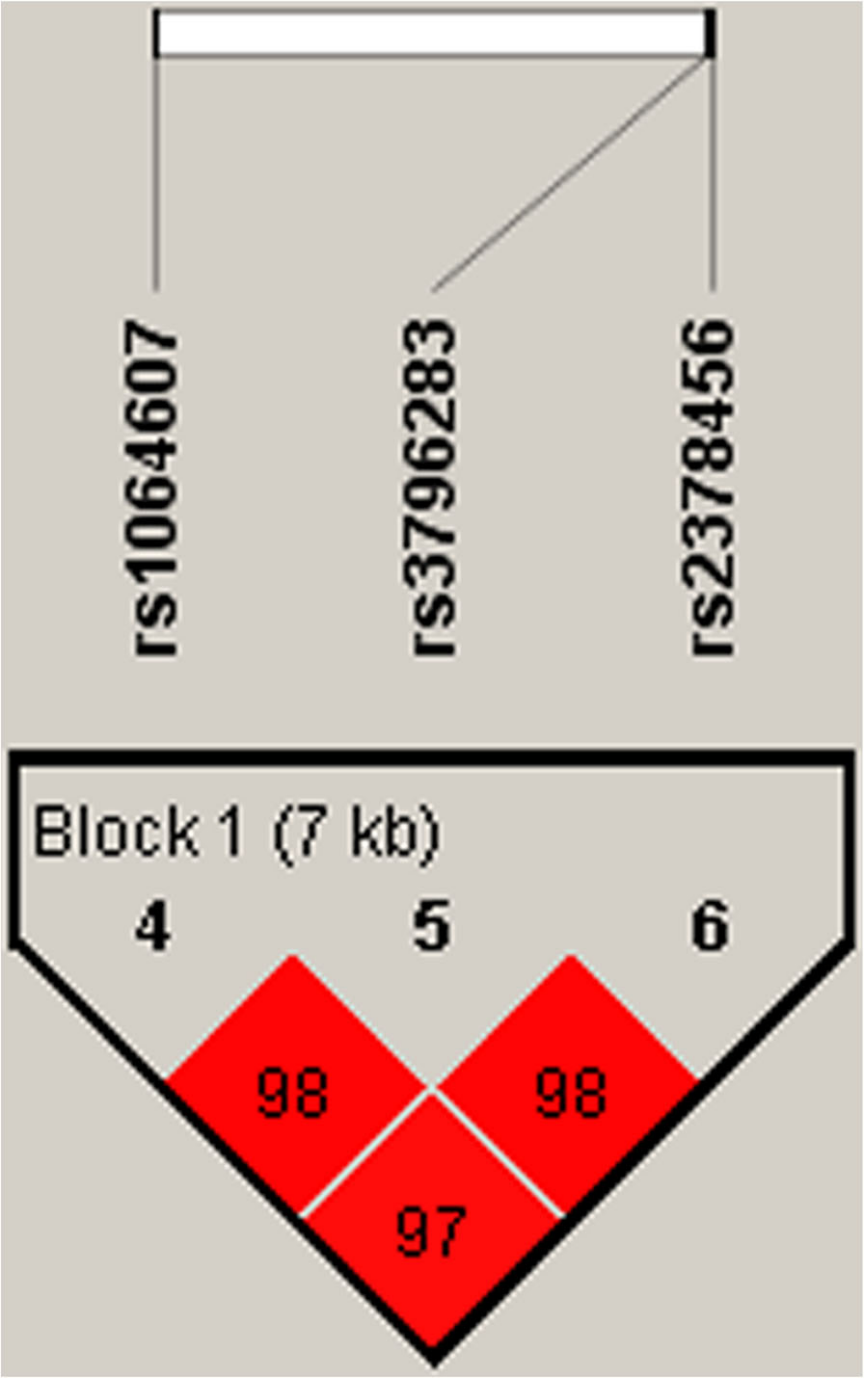
Haplotype block map for three SNPs in *LPP* gene. The numbers inside the diamonds indicate the D′ for pairwise analyses

Subsequently, haplotype analysis was used to explore the association of *LPP* gene with lung cancer susceptibility. Linkage Disequilibrium (LD) analysis demonstrated SNPs rs1064607, rs3796283 and rs2378456 composes a LD block, as shown in Fig. 1. To examine the effect of haplotypes on the risk of lung cancer, the haplotype-based logistic regression method adjusted by age and gender was carried out. However, the results did not reveal a significant association between common haplotypes and the risk of lung cancer (*p* > 0.05, Additional file 1: Table S9).

Then, MDR was used to analyze the interactions of these SNPs in *LPP*. Table 7 summarizes the results of MDR for analyzing interactions of these SNPs in their influences on risk of lung cancer. The best model was between rs1064607and rs2378456 with a testing accuracy of 0.513 and a maximum CVC of 10 out of 10 followed by a statistical significance of *p* < 0.05.

**Table 7:** SNP–SNP interaction models analyzed by the MDR method

Model	Training Bal. Acc.	Testing Bal. Acc.	CVC	OR (95% CI)	*p*
rs1064607	0.533	0.509	6/10	1.31 (0.96–1.79)	0.080
rs1064607, rs2378456	0.539	0.513	10/10	**1.37 (1.01–1.87)**	**0.042**
rs1064607, rs3796283, rs2378456	0.545	0.498	10/10	**1.43 (1.05–1.93)**	**0.022**

*MDR* multifactor dimensionality reduction, *Bal. Acc.* Balanced accuracy, *CVC* Cross–validation consistency, *OR* odds ratio, *95% CI* 95% confidence interval. *p* values were calculated using χ^2^ tests

Bold indicates statistical signifcance (*p *< 0.05)

## Discussions

We genotyped the seven 3′UTR SNPs in *NR5A2*, *LPP*, and *TNS3* to determine the potential association with the susceptibility to lung cancer. Noticeably, *LPP* polymorphisms (rs1064607, rs3796283 and rs2378456) were associated with increased susceptibility to lung cancer in males. *LPP* rs1064607 polymorphism was significantly correlated with increased risk of lung adenocarcinoma. Moreover, *TNS3* rs9876 polymorphism was associated with the lymphatic metastasis of lung cancer patients. To our best knowledge, this is the first study to explore the relationship between these 3′-UTRpolymorphisms and lung cancer risk in Chinese Han population. Our findings further highlight the biological significance of the genetic variations in 3′-UTR region, which may play an important role in the development of lung cancer.

*LPP* is located on chromosome 3q27.3-q28 belongs to the zyxin family of LIM domain proteins that involved in cell-cell adhesion, cell migration or invasion, cell-substrate cytoskeletal interactions, and tumorigenesis [18]. Accumulating evidence suggests that the dysregulation of *LPP* expression is associated with various types of cancers, such as breast cancer, myeloma and lung cancer [15, 19, 20]. For example, Kang et al. [21] have demonstrated the *LPP* overexpression in non-small-cell lung cancer. In addition, LPP degraded N-cadherin during lung cancer, and loss of LPP in advanced-stage of cancer may trigger further dissemination and distant metastasis of lung adenocarcinoma [20]. *TNS3* is located on chromosome 7p12.3, which encodes TNS3, an intracellular cytoskeletal-interacting protein that regulates cell motility and migration by anchoring actin to integrins [22]. Endogenous TNS3 contributes to cell migration, anchorage-independent growth, and tumorigenesis in cell lines derived from advanced lung cancer [23]. Thus, *LPP* and TNS3 may have an important role of in the tumorigenesis and progression of lung cancer.

3′UTR polymorphisms may affect the binding of miRNA to target genes and exert effects on genes expressions and tumorigenesis [24, 25]. In this study, we found that three polymorphisms (rs1064607, rs3796283, and rs2378456) in the 3′-UTR of *LPP* and TNS3 rs9876 polymorphism were significantly associated with susceptibility to lung cancer. These SNPs were identified to putatively affects the binding sites of miR-144 and miR-182, whose abnormal expression in lung cancer was associated with the proliferation, migration, and invasion of tumor cells [26, 27]. We, therefore, propose that These SNPs may affect *LPP* or *TNS3* expression in lung cancer by differential mRNA stability and binding activity of miR-144 and miR-182. While the functional relevance of this polymorphism has not yet been elucidated, our results might partially suggest a functional correlation between these polymorphisms and the risk of lung cancer, which may provide preliminary evidence of biological plausibility for the observed association in the current study.

Our study does have some limitations. First, the results of gene-to-environment interactions between *LPP* gene and lung cancer could not be obtained due to a lack of relevant information. Second, these SNPs located in the miRNA binding site were identified based on in silico analysis only; thus, additional studies are necessary to characterize the molecular mechanisms underlying the potential association with lung cancer development and progression.

## Conclusions

In summary, we provided evidence that SNPs in the 3′-UTR region of *LPP* gene may have an effect on individual susceptibility to lung cancer among Chinese Han individuals, particularly males.

## Additional files

Additional file 1: **Table S1.** Relationship between selected 3′UTR polymorphisms and risk of lung cancer according to multiple inheritance models. **Table S2**. The relationship of selected 3′UTR polymorphisms with lung cancer according to the gender stratification. **Table S3.** The relationship of selected 3′UTR polymorphisms with lung cancer according to the age stratification. **Table S4.** Relationship between selected 3′UTR polymorphisms and risk of lung adenocarcinoma. **Table S5.** Relationship between selected 3′UTR polymorphisms and risk of lung squamous cell carcinoma. **Table S6.** Relationship between selected 3′UTR polymorphisms and risk of lung small cell carcinoma. **Table S7.** Relationship of clinical stage with selected 3′UTR polymorphisms in lung cancer patients. **Table S8**. Relationship of lymphatic metastatic status with 3′UTR polymorphisms in lung cancer patients. **Table S9.** Haplotype frequencies and their associations with lung cancer risk. (DOCX 130 kb)

## Abbreviations

CIs: Confidence intervals
EMT: Epithelial-mesenchymal transition
HMGA2: High Mobility Group AT-Hook2
HWE: Hardy-Weinberg equilibrium
LPP: Lipoma preferred partner/LIM domain containing preferred translocation partner in lipoma
MAF: Minor allele frequency
NR5A2: Nuclear receptor subfamily 5 group A member 2
NSCLC: Non-small-cell lung cancer
OR: Odds ratio
SNPs: Single nucleotide polymorphisms
TNS3: Tensin3
UTR: 3′untranslated region

## References

[1] Bray F, Ferlay J, Soerjomataram I, Siegel RL, Torre LA, Jemal A (2018). Global Cancer statistics 2018: GLOBOCAN estimates of incidence and mortality worldwide for 36 cancers in 185 countries. CA Cancer J Clin.

[2] Chen W, Zheng R, Baade PD, Zhang S, Zeng H, Bray F (2016). Cancer statistics in China, 2015. CA Cancer J Clin.

[3] Wood DE, Eapen GA, Ettinger DS, Hou L, Jackman D, Kazerooni E (2012). Lung cancer screening. Journal of the National Comprehensive Cancer Network. Jnccn.

[4] Malhotra J, Malvezzi M, Negri E, La Vecchia C, Boffetta P (2016). Risk factors for lung cancer worldwide. Eur Respir J.

[5] Wang T, Chen T, Thakur A, Liang Y, Gao L, Zhang S (2015). Association of PSMA4 polymorphisms with lung cancer susceptibility and response to cisplatin-based chemotherapy in a Chinese Han population. Tumour Biol.

[6] Xun X, Wang H, Yang H, Wang H, Wang B, Kang L (2014). CLPTM1L genetic polymorphisms and interaction with smoking and alcohol drinking in lung Cancer risk: a case–control study in the Han population from Northwest China. Medicine.

[7] Hu QY, Jin TB, Wang L, Zhang L, Geng T, Liang G (2014). Genetic variation in the TP63 gene is associated with lung cancer risk in the Han population. Tumor Biol.

[8] Yamaguchi H, Wyckoff J, Condeelis J (2005). Cell migration in tumors. Curr Opin Cell Biol.

[9] Leung CS, Yeung TL, Yip KP, Wong KK, Ho SY, Mangala LS (2017). Cancer-associated fibroblasts regulate endothelial adhesion protein LPP to promote ovarian cancer chemoresistance. J Clin Investig.

[10] Ngan E, Northey JJ, Brown CM, Ursinisiegel J, Siegel PM (2013). A complex containing LPP and alpha-Actinin mediates TGFbeta-induced migration and invasion of ErbB2-expressing breast cancer cells. J Cell Sci.

[11] Shinchi Y, Hieda M, Nishioka Y, Matsumoto A, Yokoyama Y, Kimura H (2015). SUV420H2 suppresses breast cancer cell invasion through down regulation of the SH2 domain-containing focal adhesion protein tensin-3. Exp Cell Res.

[12] Nadolny C, Dong X (2015). Liver receptor homolog-1 (LRH-1): a potential therapeutic target for cancer. Cancer Biol Ther.

[13] Vess A, Blache U, Leitner L, Kurz ARM, Ehrenpfordt A, Sixt M, et al. A dual phenotype of MDA-MB-468 cancer cells reveals mutual regulation of tensin3 and adhesion plasticity. Journal of cell science 2017;130:2172-84. 10.1242/jcs.200899 28515231

[14] Zhang X, Gu D, Du M, Wang M, Cao C, Shen L (2014). Associations of NR5A2 gene polymorphisms with the clinicopathological characteristics and survival of gastric cancer. Int J Mol Sci.

[15] Li Z, Wong KY, Chan GC, Chim CSZ (2017). Epigenetic silencing of LPP/miR-28 in multiple myeloma. J Clin Pathol.

[16] Kertesz M, Iovino N, Unnerstall U, Gaul U, Segal E (2007). The role of site accessibility in microRNA target recognition. Nat Genet.

[17] Pirooz HJ, Jafari N, Rastegari M, Fathi-Roudsari M, Tasharrofi N, Shokri G (2017). Functional SNP in microRNA-491-5p binding site of MMP9 3'-UTR affects Cancer susceptibility. J Cell Biochem.

[18] Van Itallie CM, Tietgens AJ, Aponte A, Fredriksson K, Fanning AS, Gucek M (2014). Biotin ligase tagging identifies proteins proximal to E-cadherin, including lipoma preferred partner, a regulator of epithelial cell-cell and cell-substrate adhesion. J Cell Sci.

[19] Ngan E, Stoletov K, Smith HW, Common J, Muller WJ, Lewis JD (2017). LPP is a Src substrate required for invadopodia formation and efficient breast cancer lung metastasis. Nat Commun.

[20] Kuriyama S, Yoshida M, Yano S, Aiba N, Kohno T, Minamiya Y (2015). LPP inhibits collective cell migration during lung cancer dissemination. Oncogene.

[21] Ji UK, Sun HK, Kwon KC, Park JW, Jin MK (2009). Identification of novel candidate target genes, including EPHB3, MASP1 and SST at 3q26.2–q29 in squamous cell carcinoma of the lung. BMC Cancer.

[22] Carter JA, Gorecki DC, Mein CA, Ljungberg B, Hafizi S (2013). CpG dinucleotide-specific hypermethylation of the TNS3 gene promoter in human renal cell carcinoma. Epigenetics.

[23] Qian X, Li G, Vass WC, Papageorge A, Walker RC, Asnaghi L (2009). The Tensin-3 protein, including its SH2 domain, is phosphorylated by Src and contributes to tumorigenesis and metastasis. Cancer Cell.

[24] Ryan BM, Robles AI, Harris CC (2010). Genetic variation in microRNA networks: the implications for cancer research. Nat Rev Cancer.

[25] Chin LJ, Ratner E, Leng S, Zhai R, Nallur S, Babar I (2008). A SNP in a let-7 microRNA complementary site in the KRAS 3′ untranslated region increases non-small cell lung cancer risk. Cancer Res.

[26] Chen S, Li P, Li J, Wang Y, Du Y, Chen X (2015). MiR-144 inhibits proliferation and induces apoptosis and autophagy in lung Cancer cells by targeting TIGAR. Cell Physiol Biochem.

[27] Yanyan H, Chen DD, He JY, Liu XG, Zhu W, Zhang Y (2011). Overexpression of members of the microRNA-183 family is a risk factor for lung cancer: a case control study. BMC Cancer.

