# Supplementary material for: Single nucleotide polymorphism in the 3′ untranslated region of LPP is a risk factor for lung cancer: a case-control study
Source: BMC Cancer. 2019 Jan 8;19:35. doi: 10.1186/s12885-018-5241-5 (PMC6325744; doi:10.1186/s12885-018-5241-5)
Supplement: Supplementary file 1 — Table S1. Relationship between selected 3′UTR polymorphisms and risk of lung cancer according to multiple inheritance models. Table S2. The relationship of selected 3′UTR polymorphisms with lung cancer according to the gender stratification. Table S3. The relationship of selected 3′UTR polymorphisms with lung cancer according to the age stratification. Table S4. Relationship between selected 3′UTR polymorphisms and risk of lung adenocarcinoma. Table S5. Relationship between selected 3′UTR polymorphisms and risk of lung squamous cell carcinoma. Table S6. Relationship between selected 3′UTR polymorphisms and risk of lung small cell carcinoma. Table S7. Relationship of clinical stage with selected 3′UTR polymorphisms in lung cancer patients. Table S8. Relationship of lymphatic metastatic status with 3′UTR polymorphisms in lung cancer patients. Table S9. Haplotype frequencies and their associations with lung cancer risk. (DOCX 130 kb) [file 12885_2018_5241_MOESM1_ESM.docx]

# Additional file 1: Table S1 Relationship between selected 3′UTR polymorphisms and risk of lung cancer according to multiple inheritance models

| SNP | Model | Genotype | Controls | Cases | Crude analysis | | Adjusted by age and gender | |
| --- | --- | --- | --- | --- | --- | --- | --- | --- |
|  |  |  |  |  | OR (95% CI) | *p*-value | OR (95% CI) | *p*-value |
| rs2246209 | Dominant | G/G | 184 (47.9%) | 160 (49.7%) | 1.00 | 0.640 | 1.00 | 0.820 |
|  |  | G/A-A/A | 200 (52.1%) | 162 (50.3%) | 0.93 (0.69-1.25) |  | 0.96 (0.70-1.32) |  |
|  | Recessive | G/G-G/A | 344 (89.6%) | 290 (90.1%) | 1.00 | 0.830 | 1.00 | 0.740 |
|  |  | A/A | 40 (10.4%) | 32 (9.9%) | 0.95 (0.58-1.55) |  | 0.92 (0.55-1.54) |  |
|  | Log-additive | --- | --- | --- | 0.95 (0.76-1.19) | 0.650 | 0.96 (0.76-1.22) | 0.750 |
| rs1056426 | Dominant | T/T | 222 (57.8%) | 197 (61.6%) | 1.00 | 0.310 | 1.00 | 0.240 |
|  |  | C/T-C/C | 162 (42.2%) | 123 (38.4%) | 0.86 (0.63-1.16) |  | 0.82 (0.60-1.14) |  |
|  | Recessive | T/T-C/T | 363 (94.5%) | 299 (93.4%) | 1.00 | 0.540 | 1.00 | 0.490 |
|  |  | C/C | 21 (5.5%) | 21 (6.6%) | 1.21 (0.65-2.27) |  | 1.26 (0.65-2.43) |  |
|  | Log-additive | --- | --- | --- | 0.93 (0.73-1.19) | 0.560 | 0.91 (0.70-1.18) | 0.490 |
| rs1064607 | Dominant | G/G | 157 (40.9%) | 111 (34.6%) | 1.00 | 0.085 | 1.00 | 0.130 |
|  |  | G/C-C/C | 227 (59.1%) | 210 (65.4%) | 1.31 (0.96-1.78) |  | 1.29 (0.93-1.79) |  |
|  | Recessive | G/G-G/C | 331 (86.2%) | 271 (84.4%) | 1.00 | 0.510 | 1.00 | 0.340 |
|  |  | C/C | 53 (13.8%) | 50 (15.6%) | 1.15 (0.76-1.75) |  | 1.24 (0.80-1.95) |  |
|  | Log-additive | --- | --- | --- | 1.19 (0.96-1.47) | 0.120 | 1.20 (0.96-1.52) | 0.120 |
| rs3796283 | Dominant | A/A | 134 (34.9%) | 94 (29.3%) | 1.00 | 0.110 | 1.00 | 0.240 |
|  |  | G/A-G/G | 250 (65.1%) | 227 (70.7%) | 1.29 (0.94-1.78) |  | 1.23 (0.87-1.73) |  |
|  | Recessive | A/A-G/A | 316 (82.3%) | 261 (81.3%) | 1.00 | 0.740 | 1.00 | 0.790 |
|  |  | G/G | 68 (17.7%) | 60 (18.7%) | 1.07 (0.73-1.57) |  | 1.06 (0.70-1.59) |  |
|  | Log-additive | --- | --- | --- | 1.15 (0.93-1.42) | 0.210 | 1.12 (0.89-1.40) | 0.350 |
| rs2378456 | Dominant | G/G | 117 (30.6%) | 88 (27.9%) | 1.00 | 0.430 | 1.00 | 0.640 |
|  |  | G/C-C/C | 266 (69.5%) | 228 (72.2%) | 1.14 (0.82-1.58) |  | 1.09 (0.77-1.54) |  |
|  | Recessive | G/G-G/C | 302 (78.8%) | 245 (77.5%) | 1.00 | 0.670 | 1.00 | 0.770 |
|  |  | C/C | 81 (21.1%) | 71 (22.5%) | 1.08 (0.75-1.55) |  | 1.06 (0.72-1.56) |  |
|  | Log-additive | --- | --- | --- | 1.08 (0.88-1.34) | 0.460 | 1.06 (0.84-1.32) | 0.640 |
| rs3750163 | Dominant | G/G | 325 (84.6%) | 270 (83.8%) | 1.00 | 0.780 | 1.00 | 0.980 |
|  |  | G/A-A/A | 59 (15.4%) | 52 (16.1%) | 1.06 (0.71-1.59) |  | 0.99 (0.65-1.53) |  |
|  | Recessive | G/G-G/A | 382 (99.5%) | 318 (98.8%) | 1.00 | 0.300 | 1.00 | 0.170 |
|  |  | A/A | 2 (0.5%) | 4 (1.2%) | 2.40 (0.44-13.20) |  | 3.28 (0.56-19.10) |  |
|  | Log-additive | --- | --- | --- | 1.10 (0.76-1.60) | 0.610 | 1.06 (0.71-1.58) | 0.770 |
| rs9876 | Dominant | G/G | 108 (28.1%) | 86 (26.8%) | 1.00 | 0.690 | 1.00 | 0.760 |
|  |  | A/G-A/A | 276 (71.9%) | 235 (73.2%) | 1.07 (0.77-1.49) |  | 1.06 (0.74-1.50) |  |
|  | Recessive | G/G-A/G | 286 (74.5%) | 251 (78.2%) | 1.00 | 0.250 | 1.00 | 0.280 |
|  |  | A/A | 98 (25.5%) | 70 (21.8%) | 0.81 (0.57-1.16) |  | 0.81 (0.56-1.18) |  |
|  | Log-additive | --- | --- | --- | 0.95 (0.78-1.17) | 0.660 | 0.95 (0.76-1.18) | 0.650 |

SNP: single nucleotide polymorphism; OR: odds ratio; 95% CI: confidence interval.

*p* < 0.05 indicates statistical significance.

# Additional file 1: Table S2 The relationship of selected 3′UTR polymorphisms with lung cancer according to the gender stratification

| **SNP ID** | **Model** | **Genotype** | **Male** | | | | | | **Female** | | | | | |
| --- | --- | --- | --- | --- | --- | --- | --- | --- | --- | --- | --- | --- | --- | --- |
|  |  |  | **Control** | **Case** | **Adjusted analysis** | | **Crude analysis** | | **Control** | **Case** | **Adjusted analysis** | | **Crude analysis** | |
|  |  |  |  |  | **OR (95%CI)** | ***p*** | **OR (95%CI)** | ***p*** |  |  | **OR (95%CI)** | ***p*** | **OR (95%CI)** | ***p*** |
| rs2246209 | Allele | G | 386 (69.4%) | 337 (68.8%) | 1.00 | 0.820 |  |  | 142 (67.0%) | 113 (73.4%) | 1.00 | 0.190 |  |  |
|  |  | A | 170 (30.6%) | 153 (31.2%) | 1.03 (0.79-1.34 ) |  |  |  | 70 (33.0%) | 41 (26.6%) | 0.74 (0.47-1.16) |  |  |  |
|  | Codominant | G/G | 138 (49.6%) | 117 (47.8%) | 1.00 | 0.800 | 1.00 |  | 46 (43.4%) | 43 (55.8%) | 1.00 | 0.310 | 1.00 |  |
|  |  | G/A | 110 (39.6%) | 103 (42.0%) | 1.14 (0.76-1.70) |  | 1.10 (0.77-1.59) | 0.850 | 50 (47.2%) | 27 (35.1%) | 0.61 (0.32-1.16) |  | 0.58 (0.31-1.08) | 0.220 |
|  |  | A/A | 30 (10.8%) | 25 (10.2%) | 1.01 (0.54-1.91) |  | 0.98 (0.55-1.76) |  | 10 (9.4%) | 7 (9.1%) | 0.72 (0.25-2.08) |  | 0.75 (0.26-2.14) |  |
|  | Dominant | G/G | 138 (49.6%) | 117 (47.8%) | 1.00 | 0.580 | 1.00 | 0.670 | 46 (43.4%) | 43 (55.8%) | 1.00 | 0.130 | 1.00 | 0.096 |
|  |  | G/A-A/A | 140 (50.4%) | 128 (52.2%) | 1.11 (0.76-1.62) |  | 1.08 (0.76-1.52) |  | 60 (56.6%) | 34 (44.2%) | 0.63 (0.35-1.15) |  | 0.61 (0.34-1.10) |  |
|  | Recessive | G/G-G/A | 248 (89.2%) | 220 (89.8%) | 1.00 | 0.880 | 1.00 | 0.830 | 96 (90.6%) | 70 (90.9%) | 1.00 | 0.830 | 1.00 | 0.940 |
|  |  | A/A | 30 (10.8%) | 25 (10.2%) | 0.95 (0.52-1.75) |  | 0.94 (0.54-1.65) |  | 10 (9.4%) | 7 (9.1%) | 0.90 (0.32-2.49) |  | 0.96 (0.35-2.65) |  |
|  | Log-additive | --- | --- | --- | 1.05 (0.79-1.39) | 0.740 | 1.03 (0.80-1.33) | 0.820 | --- | --- | 0.75 (0.47-1.19) | 0.210 | 0.74 (0.46-1.17) | 0.190 |
| rs1056426 | Allele | T | 421 (75.7%) | 374 (77.0%) | 1.00 | 0.640 |  |  | 164 (77.4%) | 122 (79.2%) | 1.00 | 0.670 |  |  |
|  |  | C | 135 (24.3%) | 112 (23.0%) | 0.93 (0.70-1.24) |  |  |  | 48 (22.6%) | 32 (20.8%) | 0.90 (0.54-1.48) |  |  |  |
|  | Codominant | T/T | 160 (57.5%) | 147 (60.5%) | 1.00 | 0.480 | 1.00 |  | 62 (58.5%) | 50 (64.9%) | 1.00 | 0.380 | 1.00 |  |
|  |  | C/T | 101 (36.3%) | 80 (32.9%) | 0.79 (0.52-1.18) |  | 0.86 (0.60-1.25) | 0.720 | 40 (37.7%) | 22 (28.6%) | 0.69 (0.36-1.32) |  | 0.68 (0.36-1.29) | 0.350 |
|  |  | C/C | 17 (6.1%) | 16 (6.6%) | 1.05 (0.49-2.27) |  | 1.02 (0.50-2.10) |  | 4 (3.8%) | 5 (6.5%) | 1.55 (0.39-6.15) |  | 1.55 (0.40-6.08) |  |
|  | Dominant | T/T | 160 (57.5%) | 147 (60.5%) | 1.00 | 0.320 | 1.00 | 0.500 | 62 (58.5%) | 50 (64.9%) | 1.00 | 0.410 | 1.00 | 0.380 |
|  |  | C/T-C/C | 118 (42.5%) | 96 (39.5%) | 0.82 (0.56-1.21) |  | 0.89 (0.62-1.26) |  | 44 (41.5%) | 27 (35.1%) | 0.77 (0.42-1.42) |  | 0.76 (0.41-1.40) |  |
|  | Recessive | T/T-C/T | 261 (93.9%) | 227 (93.4%) | 1.00 | 0.720 | 1.00 | 0.830 | 102 (96.2%) | 72 (93.5%) | 1.00 | 0.420 | 1.00 | 0.400 |
|  |  | C/C | 17 (6.1%) | 16 (6.6%) | 1.15 (0.54-2.45) |  | 1.08 (0.53-2.19) |  | 4 (3.8%) | 5 (6.5%) | 1.76 (0.45-6.86) |  | 1.77 (0.46-6.82) |  |
|  | Log-additive | --- | --- | --- | 0.90 (0.67-1.23) | 0.520 | 0.94 (0.71-1.24) | 0.650 | --- | --- | 0.91 (0.54-1.50) | 0.700 | 0.90 (0.54-1.48) | 0.670 |
| rs3750163 | Allele | G | 517 (93.0%) | 445 (90.8%) | 1.00 | 0.200 |  |  | 190 (89.6%) | 143 (92.9%) | 1.00 | 0.290 |  |  |
|  |  | A | 39 (7.0%) | 45 (9.2%) | 1.34 (0.86-2.10) |  |  |  | 22 (10.4%) | 11 (7.1%) | 0.66 (0.31-1.41) |  |  |  |
|  | Codominant | G/G | 240 (86.3%) | 204 (83.3%) | 1.00 | 0.084 | 1.00 |  | 85 (80.2%) | 66 (85.7%) | 1.00 | 0.290 | 1.00 |  |
|  |  | G/A | 37 (13.3%) | 37 (15.1%) | 1.12 (0.65-1.91) |  | 1.18 (0.72-1.93) | 0.250 | 20 (18.9%) | 11 (14.3%) | 0.65 (0.29-1.47) |  | 0.71 (0.32-1.58) | 0.400 |
|  |  | A/A | 1 (0.4%) | 4 (1.6%) | 9.26 (0.97-88.21) |  | 4.71 (0.52-42.39) |  | 1 (0.9%) | 0 (0%) | 0.00 (0.00-NA) |  | 0.00 (0.00-NA) |  |
|  | Dominant | G/G | 240 (86.3%) | 204 (83.3%) | 1.00 | 0.360 | 1.00 | 0.330 | 85 (80.2%) | 66 (85.7%) | 1.00 | 0.230 | 1.00 | 0.330 |
|  |  | G/A-A/A | 38 (13.7%) | 41 (16.7%) | 1.28 (0.76-2.15) |  | 1.27 (0.79-2.05) |  | 21 (19.8%) | 11 (14.3%) | 0.61 (0.27-1.38) |  | 0.67 (0.30-1.50) |  |
|  | Recessive | G/G-G/A | 277 (99.6%) | 241 (98.4%) | 1.00 | 0.029 | 1.00 | 0.130 | 105 (99.1%) | 77 (100.0%) | 1.00 | 0.250 | 1.00 | 0.300 |
|  |  | A/A | 1 (0.4%) | 4 (1.6%) | 9.11 (0.96-86.67) |  | 4.60 (0.51-41.37) |  | 1 (0.9%) | 0 (0.0%) | 0.00 (0.00-NA) |  | 0.00 (0.00-NA) |  |
|  | Log-additive | --- | --- | --- | 1.40 (0.87-2.24) | 0.170 | 1.33 (0.85-2.06) | 0.210 | --- | --- | 0.59 (0.27-1.30) | 0.180 | 0.65 (0.30-1.41) | 0.270 |
| rs9876 | Allele | G | 279 (50.2%) | 229 (46.9%) | 1.00 | 0.300 |  |  | 117 (55.2%) | 78 (50.6%) | 1.00 | 0.390 |  |  |
|  |  | A | 277 (49.8%) | 259 (53.1%) | 1.14 (0.89-1.45) |  |  |  | 95 (44.8%) | 76 (49.4%) | 1.20 (0.79-1.82) |  |  |  |
|  | Codominant | G/G | 74 (26.6%) | 66 (27.1%) | 1.00 | 0.290 | 1.00 |  | 34 (32.1%) | 20 (26%) | 1.00 | 0.590 | 1.00 |  |
|  |  | A/G | 129 (46.4%) | 127 (52%) | 1.03 (0.65-1.62) |  | 1.10 (0.73-1.67) | 0.240 | 49 (46.2%) | 38 (49.4%) | 1.38 (0.68-2.80) |  | 1.32 (0.66-2.64) | 0.660 |
|  |  | A/A | 75 (27.0%) | 51 (20.9%) | 0.71 (0.42-1.22) |  | 0.76 (0.47-1.24) |  | 23 (21.7%) | 19 (24.7%) | 1.45 (0.63-3.33) |  | 1.40 (0.62-3.19) |  |
|  | Dominant | G/G | 74 (26.6%) | 66 (27.1%) | 1.00 | 0.680 | 1.00 | 0.910 | 34 (32.1%) | 20 (26.0%) | 1.00 | 0.310 | 1.00 | 0.370 |
|  |  | A/G-A/A | 204 (73.4%) | 178 (73%) | 0.91 (0.60-1.40) |  | 0.98 (0.66-1.44) |  | 72 (67.9%) | 57 (74.0%) | 1.40 (0.73-2.72) |  | 1.35 (0.70-2.58) |  |
|  | Recessive | G/G-A/G | 203 (73.0%) | 193 (79.1%) | 1.00 | 0.120 | 1.00 | 0.100 | 83 (78.3%) | 58 (75.3%) | 1.00 | 0.630 | 1.00 | 0.640 |
|  |  | A/A | 75 (27.0%) | 51 (20.9%) | 0.70 (0.45-1.09) |  | 0.72 (0.48-1.07) |  | 23 (21.7%) | 19 (24.7%) | 1.19 (0.59-2.39) |  | 1.18 (0.59-2.37) |  |
|  | Log-additive | --- | --- | --- | 0.85 (0.65-1.11) | 0.230 | 0.88 (0.69-1.12) | 0.300 | --- | --- | 1.21 (0.80-1.83) | 0.360 | 1.19 (0.79-1.79) | 0.400 |

SNP: single nucleotide polymorphism; OR: odds ratio; 95% CI: confidence interval. *p* < 0.05 indicates statistical significance.

# Additional file 1: Table S3 The relationship of selected 3′UTR polymorphisms with lung cancer according to the age stratification

| **SNP ID** | **Model** | **Genotype** | **≥ 50 years** | | | | | | **< 50 years** | | | | | |
| --- | --- | --- | --- | --- | --- | --- | --- | --- | --- | --- | --- | --- | --- | --- |
|  |  |  | **Control** | **Case** | **Adjusted analysis** | | **Crude analysis** | | **Control** | **Case** | **Adjusted analysis** | | **Crude analysis** | |
|  |  |  |  |  | **OR (95%CI)** | ***p*** | **OR (95%CI)** | ***p*** |  |  | **OR (95%CI)** | ***p*** | **OR (95%CI)** | ***p*** |
| rs2246209 | Allele | G | 318 (69.7%) | 366 (69.3%) | 1.00 | 0.890 |  |  | 210 (67.3%) | 84 (72.4%) | 1.00 | 0.310 |  |  |
|  |  | A | 138 (30.3%) | 162 (30.7%) | 1.02 (0.78-1.34) |  |  |  | 102 (32.7%) | 32 (27.6%) | 0.78 (0.49-1.26) |  |  |  |
|  | Codominant | G/G | 115 (50.4%) | 131 (49.6%) | 1.00 | 0.980 | 1.00 |  | 69 (44.2%) | 29 (50.0%) | 1.00 | 0.610 | 1.00 |  |
|  |  | G/A | 88 (38.6%) | 104 (39.4%) | 1.03 (0.69-1.53) |  | 1.04 (0.71-1.51) | 0.980 | 72 (46.1%) | 26 (44.8%) | 0.91 (0.47-1.77) |  | 0.86 (0.46-1.60) | 0.490 |
|  |  | A/A | 25 (11.0%) | 29 (11.0%) | 1.06 (0.58-1.96) |  | 1.02 (0.56-1.84) |  | 15 (9.6%) | 3 (5.2%) | 0.52 (0.13-2.04) |  | 0.48 (0.13-1.77) |  |
|  | Dominant | G/G | 115 (50.4%) | 131 (49.6%) | 1.00 | 0.850 | 1.00 | 0.860 | 69 (44.2%) | 29 (50.0%) | 1.00 | 0.610 | 1.00 | 0.450 |
|  |  | G/A-A/A | 113 (49.6%) | 133 (50.4%) | 1.04 (0.72-1.50) |  | 1.03 (0.72-1.47) |  | 87 (55.8%) | 29 (50.0%) | 0.84 (0.44-1.61) |  | 0.79 (0.43-1.45) |  |
|  | Recessive | G/G-G/A | 203 (89.0%) | 235 (89.0%) | 1.00 | 0.870 | 1.00 | 0.990 | 141 (90.4%) | 55 (94.8%) | 1.00 | 0.340 | 1.00 | 0.280 |
|  |  | A/A | 25 (11.0%) | 29 (11.0%) | 1.05 (0.58-1.89) |  | 1.00 (0.57-1.77) |  | 15 (9.6%) | 3 (5.2%) | 0.54 (0.14-2.06) |  | 0.51 (0.14-1.84) |  |
|  | Log-additive | --- | --- | --- | 1.03 (0.79-1.35) | 0.830 | 1.02 (0.78-1.32) | 0.890 | --- | --- | 0.81 (0.48-1.36) | 0.420 | 0.77 (0.47-1.26) | 0.290 |
| rs1056426 | Allele | T | 349 (76.5%) | 402 (76.7%) | 1.00 | 0.950 |  |  | 236 (75.6%) | 94 (81.0%) | 1.00 | 0.240 |  |  |
|  |  | C | 107 (23.5%) | 122 (23.3%) | 0.99 (0.74-1.33) |  |  |  | 76 (24.4%) | 22 (19.0%) | 0.73 (0.43-1.24) |  |  |  |
|  | Codominant | T/T | 134 (58.8%) | 159 (60.7%) | 1.00 | 0.370 | 1.00 |  | 88 (56.4%) | 38 (65.5%) | 1.00 | 0.380 | 1.00 |  |
|  |  | C/T | 81 (35.5%) | 84 (32.1%) | 0.80 (0.54-1.20) |  | 0.87 (0.60-1.28) | 0.620 | 60 (38.5%) | 18 (31.0%) | 0.62 (0.31-1.24) |  | 0.69 (0.36-1.33) | 0.470 |
|  |  | C/C | 13 (5.7%) | 19 (7.2%) | 1.31 (0.61-2.82) |  | 1.23 (0.59-2.59) |  | 8 (5.1%) | 2 (3.5%) | 0.68 (0.13-3.66) |  | 0.58 (0.12-2.85) |  |
|  | Dominant | T/T | 134 (58.8%) | 159 (60.7%) | 1.00 | 0.480 | 1.00 | 0.670 | 88 (56.4%) | 38 (65.5%) | 1.00 | 0.160 | 1.00 | 0.230 |
|  |  | C/T-C/C | 94 (41.2%) | 103 (39.3%) | 0.87 (0.60-1.27) |  | 0.92 (0.64-1.33) |  | 68 (43.6%) | 20 (34.5%) | 0.62 (0.32-1.22) |  | 0.68 (0.36-1.28) |  |
|  | Recessive | T/T-C/T | 215 (94.3%) | 243 (92.8%) | 1.00 | 0.350 | 1.00 | 0.490 | 148 (94.9%) | 56 (96.5%) | 1.00 | 0.800 | 1.00 | 0.590 |
|  |  | C/C | 13 (5.7%) | 19 (7.2%) | 1.42 (0.67-3.01) |  | 1.29 (0.62-2.68) |  | 8 (5.1%) | 2 (3.5%) | 0.81 (0.15-4.28) |  | 0.66 (0.14-3.21) |  |
|  | Log-additive | --- | --- | --- | 0.97 (0.72-1.31) | 0.850 | 0.99 (0.74-1.32) | 0.950 | --- | --- | 0.69 (0.38-1.23) | 0.200 | 0.72 (0.42-1.24) | 0.220 |
| rs1064607 | Allele | G | 290 (63.6%) | 319 (60.6%) | 1.00 | 0.340 |  |  | 198 (63.5%) | 63 (54.3%) | 1.00 | 0.085 |  |  |
|  |  | C | 166 (36.4%) | 207 (39.4%) | 1.13 (0.88-1.47) |  |  |  | 114 (36.5%) | 53 (45.7%) | 1.46 (0.95-2.25) |  |  |  |
|  | Codominant | G/G | 93 (40.8%) | 93 (35.4%) | 1.00 | 0.500 | 1.00 |  | 64 (41.0%) | 18 (31.0%) | 1.00 | 0.460 | 1.00 |  |
|  |  | G/C | 104 (45.6%) | 133 (50.6%) | 1.26 (0.85-1.88) |  | 1.28 (0.87-1.88) | 0.450 | 70 (44.9%) | 27 (46.5%) | 1.18 (0.57-2.46) |  | 1.37 (0.69-2.72) | 0.240 |
|  |  | C/C | 31 (13.6%) | 37 (14.1%) | 1.23 (0.69-2.19) |  | 1.19 (0.68-2.08) |  | 22 (14.1%) | 13 (22.4%) | 1.81 (0.72-4.60) |  | 2.10 (0.89-4.98) |  |
|  | Dominant | G/G | 93 (40.8%) | 93 (35.4%) | 1.00 | 0.240 | 1.00 | 0.220 | 64 (41.0%) | 18 (31.0%) | 1.00 | 0.410 | 1.00 | 0.180 |
|  |  | G/C-C/C | 135 (59.2%) | 170 (64.6%) | 1.25 (0.86-1.83) |  | 1.26 (0.87-1.82) |  | 92 (59.0%) | 40 (69.0%) | 1.33 (0.67-2.65) |  | 1.55 (0.81-2.94) |  |
|  | Recessive | G/G-G/C | 197 (86.4%) | 226 (85.9%) | 1.00 | 0.780 | 1.00 | 0.880 | 134 (85.9%) | 45 (77.6%) | 1.00 | 0.240 | 1.00 | 0.150 |
|  |  | C/C | 31 (13.6%) | 37 (14.1%) | 1.08 (0.63-1.84) |  | 1.04 (0.62-1.74) |  | 22 (14.1%) | 13 (22.4%) | 1.65 (0.72-3.76) |  | 1.76 (0.82-3.78) |  |
|  | Log-additive | --- | --- | --- | 1.15 (0.87-1.50) | 0.330 | 1.14 (0.87-1.48) | 0.340 | --- | --- | 1.32 (0.83-2.10) | 0.230 | 1.44 (0.94-2.20) | 0.093 |
| rs3796283 | Allele | A | 264 (57.9%) | 297 (56.5%) | 1.00 | 0.650 |  |  | 186 (59.6%) | 58 (50.0%) | 1.00 | 0.074 |  |  |
|  |  | G | 192 (42.1%) | 229 (43.5%) | 1.06 (0.82-1.37) |  |  |  | 126 (40.4%) | 58 (50.0%) | 1.48 (0.96-2.27) |  |  |  |
|  | Codominant | A/A | 76 (33.3%) | 80 (30.4%) | 1.00 | 0.800 | 1.00 |  | 58 (37.2%) | 14 (24.1%) | 1.00 | 0.310 | 1.00 |  |
|  |  | G/A | 112 (49.1%) | 137 (52.1%) | 1.14 (0.75-1.73) |  | 1.16 (0.78-1.74) | 0.760 | 70 (44.9%) | 30 (51.7%) | 1.76 (0.81-3.81) |  | 1.78 (0.86-3.66) | 0.170 |
|  |  | G/G | 40 (17.5%) | 46 (17.5%) | 1.03 (0.59-1.78) |  | 1.09 (0.64-1.85) |  | 28 (17.9%) | 14 (24.1%) | 1.75 (0.68-4.48) |  | 2.07 (0.87-4.93) |  |
|  | Dominant | A/A | 76 (33.3%) | 80 (30.4%) | 1.00 | 0.600 | 1.00 | 0.490 | 58 (37.2%) | 14 (24.1%) | 1.00 | 0.120 | 1.00 | 0.068 |
|  |  | G/A-G/G | 152 (66.7%) | 183 (69.6%) | 1.11 (0.75-1.65) |  | 1.14 (0.78-1.67) |  | 98 (62.8%) | 44 (75.9%) | 1.76 (0.84-3.65) |  | 1.86 (0.94-3.68) |  |
|  | Recessive | A/A-G/A | 188 (82.5%) | 217 (82.5%) | 1.00 | 0.830 | 1.00 | 0.990 | 128 (82.0%) | 44 (75.9%) | 1.00 | 0.620 | 1.00 | 0.320 |
|  |  | G/G | 40 (17.5%) | 46 (17.5%) | 0.95 (0.58-1.54) |  | 1.00 (0.62-1.59) |  | 28 (17.9%) | 14 (24.1%) | 1.22 (0.56-2.70) |  | 1.45 (0.70-3.01) |  |
|  | Log-additive | --- | --- | --- | 1.03 (0.79-1.35) | 0.810 | 1.06 (0.82-1.38) | 0.650 | --- | --- | 1.35 (0.85-2.12) | 0.200 | 1.45 (0.95-2.22) | 0.081 |
| rs2378456 | Allele | G | 244 (53.7%) | 279 (53.7%) | 1.00 | 0.980 |  |  | 175 (56.1%) | 54 (48.2%) | 1.00 | 0.150 |  |  |
|  |  | C | 210 (46.3%) | 241 (46.3%) | 1.00 (0.78-1.29) |  |  |  | 137 (43.9%) | 58 (51.8%) | 1.37 (0.89-2.12) |  |  |  |
|  | Codominant | G/G | 65 (28.6%) | 75 (28.9%) | 1.00 | 0.980 | 1.00 |  | 52 (33.3%) | 13 (23.2%) | 1.00 | 0.470 | 1.00 |  |
|  |  | G/C | 114 (50.2%) | 129 (49.6%) | 0.97 (0.63-1.50) |  | 0.98 (0.65-1.49) | 0.990 | 71 (45.5%) | 28 (50.0%) | 1.63 (0.73-3.64) |  | 1.58 (0.75-3.34) | 0.330 |
|  |  | C/C | 48 (21.1%) | 56 (21.5%) | 0.96 (0.56-1.63) |  | 1.01 (0.61-1.68) |  | 33 (21.1%) | 15 (26.8%) | 1.49 (0.59-3.81) |  | 1.82 (0.77-4.30) |  |
|  | Dominant | G/G | 65 (28.6%) | 75 (28.9%) | 1.00 | 0.870 | 1.00 | 0.960 | 52 (33.3%) | 13 (23.2%) | 1.00 | 0.230 | 1.00 | 0.150 |
|  |  | G/C-C/C | 162 (71.4%) | 185 (71.2%) | 0.97 (0.64-1.45) |  | 0.99 (0.67-1.47) |  | 104 (66.7%) | 43 (76.8%) | 1.58 (0.74-3.37) |  | 1.65 (0.82-3.34) |  |
|  | Recessive | G/G-G/C | 179 (78.8%) | 204 (78.5%) | 1.00 | 0.910 | 1.00 | 0.920 | 123 (78.8%) | 41 (73.2%) | 1.00 | 0.820 | 1.00 | 0.390 |
|  |  | C/C | 48 (21.1%) | 56 (21.5%) | 0.98 (0.62-1.54) |  | 1.02 (0.66-1.58) |  | 33 (21.1%) | 15 (26.8%) | 1.10 (0.51-2.37) |  | 1.36 (0.67-2.76) |  |
|  | Log-additive | --- | --- | --- | 0.98 (0.75-1.27) | 0.870 | 1.00 (0.78-1.29) | 0.980 | --- | --- | 1.23 (0.78-1.94) | 0.380 | 1.35 (0.88-2.06) | 0.160 |
| rs3750163 | Allele | G | 416 (91.2%) | 484 (91.7%) | 1.00 | 0.810 |  |  | 291 (93.3%) | 104 (89.7%) | 1.00 | 0.210 |  |  |
|  |  | A | 40 (8.8%) | 44 (8.3%) | 0.95 (0.60-1.48) |  |  |  | 21 (6.7%) | 12 (10.3%) | 1.60 (0.76-3.36) |  |  |  |
|  | Codominant | G/G | 190 (83.3%) | 221 (83.7%) | 1.00 | 0.83 | 1.00 |  | 135 (86.5%) | 49 (84.5%) | 1.00 | 0.009 | 1.00 |  |
|  |  | G/A | 36 (15.8%) | 42 (15.9%) | 1.03 (0.62-1.71) |  | 1.00 (0.62-1.63) | 0.780 | 21 (13.5%) | 6 (10.3%) | 0.62 (0.22-1.72) |  | 0.79 (0.30-2.06) | 0.017 |
|  |  | A/A | 2 (0.9%) | 1 (0.4%) | 0.49 (0.04-5.50) |  | 0.43 (0.04-4.77) |  | 0 (0%) | 3 (5.2%) | NA (0.00-NA) |  | NA (0.00-NA) |  |
|  | Dominant | G/G | 190 (83.3%) | 221 (83.7%) | 1.00 | 1.00 | 1.00 | 0.910 | 135 (86.5%) | 49 (84.5%) | 1.00 | 0.990 | 1.00 | 0.70 |
|  |  | G/A-A/A | 38 (16.7%) | 43 (16.3%) | 1.00 (0.61-1.64) |  | 0.97 (0.60-1.57) |  | 21 (13.5%) | 9 (15.5%) | 1.00 (0.41-2.44) |  | 1.18 (0.51-2.75) |  |
|  | Recessive | G/G-G/A | 226 (99.1%) | 263 (99.6%) | 1.00 | 0.550 | 1.00 | 0.480 | 156 (100%) | 55 (94.8%) | 1.00 | 0.003 | 1.00 | 0.005 |
|  |  | A/A | 2 (0.9%) | 1 (0.4%) | 0.49 (0.04-5.46) |  | 0.43 (0.04-4.76) |  | 0 (0%) | 3 (5.2%) | NA (0.00-NA) |  | NA (0.00-NA) |  |
|  | Log-additive | --- | --- | --- | 0.97 (0.61-1.55) | 0.900 | 0.94 (0.60-1.48) | 0.800 | --- | --- | 1.40 (0.67-2.93) | 0.380 | 1.52 (0.75-3.09) | 0.250 |
| rs9876 | Allele | G | 238 (52.2%) | 274 (52.1%) | 1.00 | 0.970 |  |  | 156 (50.0%) | 63 (54.3%) | 1.00 | 0.430 |  |  |
|  |  | A | 218 (47.8%) | 252 (47.9%) | 1.00 (0.78-1.29) |  |  |  | 156 (50.0%) | 53 (45.7%) | 0.84 (0.55-1.29) |  |  |  |
|  | Codominant | G/G | 64 (28.1%) | 70 (26.6%) | 1.00 | 0.850 | 1.00 |  | 44 (28.2%) | 16 (27.6%) | 1.00 | 0.280 | 1.00 |  |
|  |  | A/G | 110 (48.2%) | 134 (51.0%) | 1.07 (0.69-1.67) |  | 1.11 (0.73-1.70) | 0.840 | 68 (43.6%) | 31 (53.5%) | 1.38 (0.64-2.95) |  | 1.25 (0.61-2.56) | 0.310 |
|  |  | A/A | 54 (23.7%) | 59 (22.4%) | 0.94 (0.56-1.59) |  | 1.00 (0.61-1.65) |  | 44 (28.2%) | 11 (19.0%) | 0.72 (0.29-1.82) |  | 0.69 (0.29-1.65) |  |
|  | Dominant | G/G | 64 (28.1%) | 70 (26.6%) | 1.00 | 0.880 | 1.00 | 0.720 | 44 (28.2%) | 16 (27.6%) | 1.00 | 0.770 | 1.00 | 0.930 |
|  |  | A/G-A/A | 164 (71.9%) | 193 (73.4%) | 1.03 (0.68-1.56) |  | 1.08 (0.72-1.60) |  | 112 (71.8%) | 42 (72.4%) | 1.11 (0.54-2.28) |  | 1.03 (0.53-2.02) |  |
|  | Recessive | G/G-A/G | 174 (76.3%) | 204 (77.6%) | 1.00 | 0.640 | 1.00 | 0.740 | 112 (71.8%) | 47 (81.0%) | 1.00 | 0.180 | 1.00 | 0.160 |
|  |  | A/A | 54 (23.7%) | 59 (22.4%) | 0.90 (0.58-1.40) |  | 0.93 (0.61-1.42) |  | 44 (28.2%) | 11 (19.0%) | 0.59 (0.27-1.29) |  | 0.60 (0.28-1.25) |  |
|  | Log-additive | --- | --- | --- | 0.98 (0.75-1.27) | 0.850 | 1.00 (0.78-1.29) | 0.970 | --- | --- | 0.87 (0.56-1.35) | 0.540 | 0.85 (0.56-1.29) | 0.440 |

OR, odds ratio; CI, confidence interval.

*p* < 0.05 indicates statistical significance.

# Additional file 1: Table S4 Relationship between selected 3′UTR polymorphisms and risk of lung adenocarcinoma

| SNP ID | Model | Genotype | Control | Adenocarcinoma | Adjusted analysis | | Crude analysis | |
| --- | --- | --- | --- | --- | --- | --- | --- | --- |
|  |  |  |  |  | OR (95%CI) | *p* | OR (95%CI) | *p* |
| rs2246209 | Allele | G | 528 (68.8%) | 212 (70.7%) | 1.00 | 0.542 |  |  |
|  |  | A | 240 (31.3%) | 88 (29.3%) | 0.91 (0.68-1.22) |  |  |  |
|  | Codominant | G/G | 184 (47.9%) | 78 (52.0%) | 1.00 | 0.730 | 1.00 |  |
|  |  | G/A | 160 (41.7%) | 56 (37.3%) | 0.85 (0.56-1.29) |  | 0.83 (0.55-1.24) | 0.640 |
|  |  | A/A | 40 (10.4%) | 16 (10.7%) | 0.88 (0.46-1.71) |  | 0.94 (0.50-1.78) |  |
|  | Dominant | G/G | 184 (47.9%) | 78 (52.0%) | 1.00 | 0.430 | 1.00 | 0.400 |
|  |  | G/A-A/A | 200 (52.1%) | 72 (48.0%) | 0.85 (0.58-1.27) |  | 0.85 (0.58-1.24) |  |
|  | Recessive | G/G-G/A | 344 (89.6%) | 134 (89.3%) | 1.00 | 0.880 | 1.00 | 0.930 |
|  |  | A/A | 40 (10.4%) | 16 (10.7%) | 0.95 (0.50-1.80) |  | 1.03 (0.56-1.90) |  |
|  | Log-additive | --- | --- | --- | 0.91 (0.68-1.22) | 0.510 | 0.92 (0.69-1.22) | 0.550 |
| rs1056426 | Allele | T | 585 (76.2%) | 237 (79.0%) | 1.00 | 0.324 |  |  |
|  |  | C | 183 (23.8%) | 63 (21.0%) | 0.85 (0.61-1.17) |  |  |  |
|  | Codominant | T/T | 222 (57.8%) | 96 (64.0%) | 1.00 | 0.240 | 1.00 |  |
|  |  | C/T | 141 (36.7%) | 45 (30.0%) | 0.70 (0.45-1.07) |  | 0.74 (0.49-1.11) | 0.340 |
|  |  | C/C | 21 (5.5%) | 9 (6.0%) | 0.97 (0.41-2.26) |  | 0.99 (0.44-2.24) |  |
|  | Dominant | T/T | 222 (57.8%) | 96 (64.0%) | 1.00 | 0.130 | 1.00 | 0.190 |
|  |  | C/T-C/C | 162 (42.2%) | 54 (36.0%) | 0.73 (0.49-1.10) |  | 0.77 (0.52-1.14) |  |
|  | Recessive | T/T-C/T | 363 (94.5%) | 141 (94.0%) | 1.00 | 0.820 | 1.00 | 0.810 |
|  |  | C/C | 21 (5.5%) | 9 (6.0%) | 1.10 (0.48-2.54) |  | 1.10 (0.49-2.47) |  |
|  | Log-additive | --- | --- | --- | 0.82 (0.59-1.15) | 0.250 | 0.85 (0.62-1.17) | 0.320 |
| rs1064607 | Allele | G | 488 (63.5%) | 169 (56.3%) | 1.00 | **0.030** |  |  |
|  |  | C | 280 (36.5%) | 131 (43.7%) | **1.35 (1.03-1.77)** |  |  |  |
|  | Codominant | G/G | 157 (40.9%) | 47 (31.3%) | 1.00 | 0.085 | 1.00 |  |
|  |  | G/C | 174 (45.3%) | 75 (50%) | 1.39 (0.89-2.16) |  | 1.44 (0.94-2.20) | 0.091 |
|  |  | C/C | 53 (13.8%) | 28 (18.7%) | 1.90 (1.06-3.42) |  | 1.76 (1.01-3.10) |  |
|  | Dominant | G/G | 157 (40.9%) | 47 (31.3%) | 1.00 | 0.053 | 1.00 | **0.040** |
|  |  | G/C-C/C | 227 (59.1%) | 103 (68.7%) | 1.50 (0.99-2.28) |  | **1.52 (1.02-2.26)** |  |
|  | Recessive | G/G-G/C | 331 (86.2%) | 122 (81.3%) | 1.00 | 0.096 | 1.00 | 0.170 |
|  |  | C/C | 53 (13.8%) | 28 (18.7%) | 1.57 (0.93-2.66) |  | 1.43 (0.87-2.37) |  |
|  | Log-additive | --- | --- | --- | **1.38 (1.04-1.83)** | **0.026** | **1.35 (1.03-1.77)** | **0.031** |
| rs3796283 | Allele | A | 450 (58.6%) | 159 (53.0%) | 1.00 | 0.097 |  |  |
|  |  | G | 318 (41.4%) | 141 (47.0%) | 1.25 (0.96-1.64) |  |  |  |
|  | Codominant | A/A | 134 (34.9%) | 41 (27.3%) | 1.00 | 0.350 | 1.00 |  |
|  |  | G/A | 182 (47.4%) | 77 (51.3%) | 1.31 (0.83-2.06) |  | 1.38 (0.89-2.15) | 0.220 |
|  |  | G/G | 68 (17.7%) | 32 (21.3%) | 1.47 (0.83-2.60) |  | 1.54 (0.89-2.66) |  |
|  | Dominant | A/A | 134 (34.9%) | 41 (27.3%) | 1.00 | 0.170 | 1.00 | 0.091 |
|  |  | G/A-G/G | 250 (65.1%) | 109 (72.7%) | 1.35 (0.88-2.08) |  | 1.42 (0.94-2.16) |  |
|  | Recessive | A/A-G/A | 316 (82.3%) | 118 (78.7%) | 1.00 | 0.390 | 1.00 | 0.340 |
|  |  | G/G | 68 (17.7%) | 32 (21.3%) | 1.25 (0.76-2.04) |  | 1.26 (0.79-2.02) |  |
|  | Log-additive | --- | --- | --- | 1.22 (0.92-1.62) | 0.160 | 1.25 (0.96-1.64) | 0.099 |
| rs2378456 | Allele | G | 419 (54.7%) | 153 (51.7%) | 1.00 | 0.378 |  |  |
|  |  | C | 347 (45.3%) | 143 (48.3%) | 1.13 (0.86-1.48) |  |  |  |
|  | Codominant | G/G | 117 (30.6%) | 42 (28.4%) | 1.00 | 0.700 | 1.00 | 0.630 |
|  |  | G/C | 185 (48.3%) | 69 (46.6%) | 1.01 (0.63-1.61) |  | 1.04 (0.66-1.63) |  |
|  |  | C/C | 81 (21.1%) | 37 (25.0%) | 1.23 (0.71-2.13) |  | 1.27 (0.75-2.15) |  |
|  | Dominant | G/G | 117 (30.6%) | 42 (28.4%) | 1.00 | 0.730 | 1.00 | 0.620 |
|  |  | G/C-C/C | 266 (69.5%) | 106 (71.6%) | 1.08 (0.70-1.67) |  | 1.11 (0.73-1.69) |  |
|  | Recessive | G/G-G/C | 302 (78.8%) | 111 (75.0%) | 1.00 | 0.400 | 1.00 | 0.340 |
|  |  | C/C | 81 (21.1%) | 37 (25.0%) | 1.22 (0.77-1.95) |  | 1.24 (0.80-1.94) |  |
|  | Log-additive | --- | --- | --- | 1.11 (0.84-1.46) | 0.480 | 1.12 (0.86-1.46) | 0.390 |
| rs3750163 | Allele | G | 707 (92.1%) | 274 (91.3%) | 1.00 | 0.697 |  |  |
|  |  | A | 61 (7.9%) | 26 (8.7%) | 1.10 (0.68-1.78) |  |  |  |
|  | Codominant | G/G | 325 (84.6%) | 126 (84.0%) | 1.00 | 0.650 | 1.00 |  |
|  |  | G/A | 57 (14.8%) | 22 (14.7%) | 0.93 (0.54-1.62) |  | 1.00 (0.58-1.70) | 0.650 |
|  |  | A/A | 2 (0.5%) | 2 (1.3%) | 2.57 (0.33-19.91) |  | 2.58 (0.36-18.51) |  |
|  | Dominant | G/G | 325 (84.6%) | 126 (84.0%) | 1.00 | 0.960 | 1.00 | 0.860 |
|  |  | G/A-A/A | 59 (15.4%) | 24 (16.0%) | 0.99 (0.58-1.69) |  | 1.05 (0.63-1.76) |  |
|  | Recessive | G/G-G/A | 382 (99.5%) | 148 (98.7%) | 1.00 | 0.370 | 1.00 | 0.350 |
|  |  | A/A | 2 (0.5%) | 2 (1.3%) | 2.60 (0.34-20.08) |  | 2.58 (0.36-18.49) |  |
|  | Log-additive | --- | --- | --- | 1.04 (0.64-1.71) | 0.870 | 1.10 (0.68-1.77) | 0.700 |
| rs9876 | Allele | G | 397 (51.5%) | 164 (54.7%) | 1.00 | 0.322 |  |  |
|  |  | A | 374 (48.5%) | 136 (45.3%) | 0.87 (0.67-1.14) |  |  |  |
|  | Codominant | G/G | 108 (28.1%) | 46 (30.7%) | 1.00 | 0.500 | 1.00 |  |
|  |  | A/G | 178 (46.4%) | 72 (48%) | 0.96 (0.61-1.52) |  | 0.95 (0.61-1.48) | 0.580 |
|  |  | A/A | 98 (25.5%) | 32 (21.3%) | 0.74 (0.43-1.28) |  | 0.77 (0.45-1.30) |  |
|  | Dominant | G/G | 108 (28.1%) | 46 (30.7%) | 1.00 | 0.570 | 1.00 | 0.560 |
|  |  | A/G-A/A | 276 (71.9%) | 104 (69.3%) | 0.88 (0.57-1.36) |  | 0.88 (0.59-1.34) |  |
|  | Recessive | G/G-A/G | 286 (74.5%) | 118 (78.7%) | 1.00 | 0.250 | 1.00 | 0.310 |
|  |  | A/A | 98 (25.5%) | 32 (21.3%) | 0.76 (0.47-1.22) |  | 0.79 (0.50-1.24) |  |
|  | Log-additive | --- | --- | --- | 0.87 (0.66-1.14) | 0.300 | 0.88 (0.68-1.14) | 0.340 |

OR, odds ratio; 95% CI, 95% confidence interval.

*p* ≤ 0.05 indicates statistical significance.

# Additional file 1: Table S5 Relationship between selected 3′UTR polymorphisms and risk of lung squamous cell carcinoma

| **SNP ID** | **Model** | **Genotype** | **Control** | **Squamous cell carcinoma** | **Adjusted analysis** | | **Crude analysis** | |
| --- | --- | --- | --- | --- | --- | --- | --- | --- |
|  |  |  |  |  | **OR (95%CI)** | ***p*** | **OR (95%CI)** | ***p*** |
| rs2246209 | Allele | G | 528 (68.8%) | 142 (72.4%) | 1.00 | 0.315 |  |  |
|  |  | A | 240 (31.3%) | 54 (27.6%) | 0.84 (0.59-1.19) |  |  |  |
|  | Codominant | G/G | 184 (47.9%) | 51 (52%) | 1.00 | 0.640 | 1.00 |  |
|  |  | G/A | 160 (41.7%) | 40 (40.8%) | 0.98 (0.59-1.64) |  | 0.90 (0.57-1.44) | 0.550 |
|  |  | A/A | 40 (10.4%) | 7 (7.1%) | 0.65 (0.26-1.65) |  | 0.63 (0.27-1.49) |  |
|  | Dominant | G/G | 184 (47.9%) | 51 (52%) | 1.00 | 0.710 | 1.00 | 0.470 |
|  |  | G/A-A/A | 200 (52.1%) | 47 (48%) | 0.91 (0.56-1.49) |  | 0.85 (0.54-1.32) |  |
|  | Recessive | G/G-G/A | 344 (89.6%) | 91 (92.9%) | 1.00 | 0.350 | 1.00 | 0.310 |
|  |  | A/A | 40 (10.4%) | 7 (7.1%) | 0.66 (0.27-1.62) |  | 0.66 (0.29-1.53) |  |
|  | Log-additive | --- | --- | --- | 0.87 (0.60-1.27) | 0.480 | 0.84 (0.59-1.19) | 0.320 |
| rs1056426 | Allele | T | 585 (76.2%) | 153 (78.9%) | 1.00 | 0.428 |  |  |
|  |  | C | 183 (23.8%) | 41 (21.1%) | 0.86 (0.58-1.26) |  |  |  |
|  | Codominant | T/T | 222 (57.8%) | 60 (61.9%) | 1.00 | 0.730 | 1.00 |  |
|  |  | C/T | 141 (36.7%) | 33 (34.0%) | 0.85 (0.50-1.42) |  | 0.87 (0.54-1.39) | 0.720 |
|  |  | C/C | 21 (5.5%) | 4 (4.1%) | 0.72 (0.22-2.29) |  | 0.70 (0.23-2.13) |  |
|  | Dominant | T/T | 222 (57.8%) | 60 (61.9%) | 1.00 | 0.460 | 1.00 | 0.470 |
|  |  | C/T-C/C | 162 (42.2%) | 37 (38.1%) | 0.83 (0.50-1.37) |  | 0.85 (0.54-1.33) |  |
|  | Recessive | T/T-C/T | 363 (94.5%) | 93 (95.9%) | 1.00 | 0.640 | 1.00 | 0.580 |
|  |  | C/C | 21 (5.5%) | 4 (4.1%) | 0.76 (0.24-2.40) |  | 0.74 (0.25-2.22) |  |
|  | Log-additive | --- | --- | --- | 0.85 (0.56-1.29) | 0.430 | 0.85 (0.58-1.26) | 0.420 |
| rs1064607 | Allele | G | 488 (63.5%) | 121 (62.4%) | 1.00 | 0.762 |  |  |
|  |  | C | 280 (36.5%) | 73 (37.6%) | 1.05(0.76-1.46) |  |  |  |
|  | Codominant | G/G | 157 (40.9%) | 37 (38.1%) | 1.00 | 0.760 | 1.00 |  |
|  |  | G/C | 174 (45.3%) | 47 (48.5%) | 1.22 (0.72-2.07) |  | 1.15 (0.71-1.86) | 0.850 |
|  |  | C/C | 53 (13.8%) | 13 (13.4%) | 1.13 (0.52-2.47) |  | 1.04 (0.51-2.11) |  |
|  | Dominant | G/G | 157 (40.9%) | 37 (38.1%) | 1.00 | 0.480 | 1.00 | 0.620 |
|  |  | G/C-C/C | 227 (59.1%) | 60 (61.9%) | 1.20 (0.73-1.98) |  | 1.12 (0.71-1.77) |  |
|  | Recessive | G/G-G/C | 331 (86.2%) | 84 (86.6%) | 1.00 | 0.970 | 1.00 | 0.920 |
|  |  | C/C | 53 (13.8%) | 13 (13.4%) | 1.02 (0.49-2.10) |  | 0.97 (0.50-1.86) |  |
|  | Log-additive | --- | --- | --- | 1.10 (0.77-1.58) | 0.590 | 1.05 (0.76-1.45) | 0.760 |
| rs3796283 | Allele | A | 450 (58.6%) | 110 (56.1%) | 1.00 | 0.531 |  |  |
|  |  | G | 318 (41.4%) | 86 (43.9%) | 1.11(0.81-1.52) |  |  |  |
|  | Codominant | A/A | 134 (34.9%) | 29 (29.6%) | 1.00 | 0.530 | 1.00 |  |
|  |  | G/A | 182 (47.4%) | 52 (53.1%) | 1.37 (0.79-2.39) |  | 1.32 (0.80-2.19) | 0.550 |
|  |  | G/G | 68 (17.7%) | 17 (17.4%) | 1.18 (0.56-2.46) |  | 1.16 (0.59-2.25) |  |
|  | Dominant | A/A | 134 (34.9%) | 29 (29.6%) | 1.00 | 0.300 | 1.00 | 0.320 |
|  |  | G/A-G/G | 250 (65.1%) | 69 (70.4%) | 1.32 (0.78-2.23) |  | 1.28 (0.79-2.06) |  |
|  | Recessive | A/A-G/A | 316 (82.3%) | 81 (82.7%) | 1.00 | 0.940 | 1.00 | 0.930 |
|  |  | G/G | 68 (17.7%) | 17 (17.4%) | 0.97 (0.51-1.87) |  | 0.98 (0.54-1.75) |  |
|  | Log-additive | --- | --- | --- | 1.13 (0.79-1.60) | 0.510 | 1.11 (0.81-1.52) | 0.530 |
| rs2378456 | Allele | G | 419 (54.7%) | 100 (52.1%) | 1.00 | 0.515 |  |  |
|  |  | C | 347 (45.3%) | 92 (47.9%) | 1.11(0.81-1.53) |  |  |  |
|  | Codominant | G/G | 117 (30.6%) | 24 (25.0%) | 1.00 | 0.570 | 1.00 | 0.510 |
|  |  | G/C | 185 (48.3%) | 52 (54.2%) | 1.37 (0.76-2.46) |  | 1.37 (0.80-2.34) |  |
|  |  | C/C | 81 (21.1%) | 20 (20.8%) | 1.26 (0.61-2.61) |  | 1.20 (0.62-2.32) |  |
|  | Dominant | G/G | 117 (30.6%) | 24 (25%) | 1.00 | 0.300 | 1.00 | 0.280 |
|  |  | G/C-C/C | 266 (69.5%) | 72 (75%) | 1.34 (0.77-2.33) |  | 1.32 (0.79-2.20) |  |
|  | Recessive | G/G-G/C | 302 (78.8%) | 76 (79.2%) | 1.00 | 0.920 | 1.00 | 0.950 |
|  |  | C/C | 81 (21.1%) | 20 (20.8%) | 1.03 (0.56-1.91) |  | 0.98 (0.57-1.70) |  |
|  | Log-additive | --- | --- | --- | 1.14 (0.80-1.62) | 0.470 | 1.11 (0.81-1.52) | 0.520 |
| rs3750163 | Allele | G | 707 (92.1%) | 183 (93.4%) | 1.00 | 0.539 |  |  |
|  |  | A | 61 (7.9%) | 13 (6.6%) | 0.82(0.44-1.53) |  |  |  |
|  | Codominant | G/G | 325 (84.6%) | 85 (86.7%) | 1.00 | 0.720 | 1.00 |  |
|  |  | G/A | 57 (14.8%) | 13 (13.3%) | 0.92 (0.45-1.89) |  | 0.87 (0.46-1.67) | 0.580 |
|  |  | A/A | 2 (0.5%) | 0 (0%) | / |  | / |  |
|  | Dominant | G/G | 325 (84.6%) | 85 (86.7%) | 1.00 | 0.760 | 1.00 | 0.600 |
|  |  | G/A-A/A | 59 (15.4%) | 13 (13.3%) | 0.90 (0.44-1.83) |  | 0.84 (0.44-1.61) |  |
|  | Recessive | G/G-G/A | 382 (99.5%) | 98 (100%) | 1.00 | / | 1.00 | / |
|  |  | A/A | 2 (0.5%) | 0 (0%) | / |  | / |  |
|  | Log-additive | --- | --- | --- | 0.87 (0.44-1.74) | 0.700 | 0.82 (0.44-1.54) | 0.530 |
| rs9876 | Allele | G | 397 51.5(%) | 97 (50.0%) | 1.00 | 0.746 |  |  |
|  |  | A | 374 (48.5%) | 97 (50.0%) | 1.05(0.77-1.44) |  |  |  |
|  | Codominant | G/G | 108 (28.1%) | 22 (22.7%) | 1.00 | 0.410 | 1.00 |  |
|  |  | A/G | 178 (46.4%) | 53 (54.6%) | 1.34 (0.73-2.46) |  | 1.46 (0.84-2.54) | 0.330 |
|  |  | A/A | 98 (25.5%) | 22 (22.7%) | 0.93 (0.46-1.90) |  | 1.10 (0.57-2.11) |  |
|  | Dominant | G/G | 108 (28.1%) | 22 (22.7%) | 1.00 | 0.550 | 1.00 | 0.270 |
|  |  | A/G-A/A | 276 (71.9%) | 75 (77.3%) | 1.19 (0.67-2.12) |  | 1.33 (0.79-2.25) |  |
|  | Recessive | G/G-A/G | 286 (74.5%) | 75 (77.3%) | 1.00 | 0.350 | 1.00 | 0.560 |
|  |  | A/A | 98 (25.5%) | 22 (22.7%) | 0.76 (0.43-1.36) |  | 0.86 (0.51-1.45) |  |
|  | Log-additive | --- | --- | --- | 0.97 (0.69-1.36) | 0.850 | 1.05 (0.77-1.43) | 0.750 |

OR, odds ratio; 95% CI, 95% confidence interval.

*p* ≤ 0.05 indicates statistical significance.

# Additional file 1: Table S6 Relationship between selected 3′UTR polymorphisms and risk of lung small cell carcinoma

| **SNP ID** | **Model** | **Genotype** | **Control** | **Small cell carcinoma** | **Adjusted analysis** | | **Crude analysis** | |
| --- | --- | --- | --- | --- | --- | --- | --- | --- |
|  |  |  |  |  | **OR (95%CI)** | ***p*** | **OR (95%CI)** | ***p*** |
| rs2246209 | Allele | G | 528 (68.8%) | 96 (64.9%) | 1.00 | 0.353 |  |  |
|  |  | A | 240 (31.3%) | 52 (35.1%) | 1.19 (0.82-1.73) |  |  |  |
|  | Codominant | G/G | 184 (47.9%) | 31 (41.9%) | 1.00 | 0.490 | 1.00 |  |
|  |  | G/A | 160 (41.7%) | 34 (46%) | 1.38 (0.79-2.40) |  | 1.26 (0.74-2.14) | 0.630 |
|  |  | A/A | 40 (10.4%) | 9 (12.2%) | 1.35 (0.58-3.15) |  | 1.34 (0.59-3.02) |  |
|  | Dominant | G/G | 184 (47.9%) | 31 (41.9%) | 1.00 | 0.230 | 1.00 | 0.340 |
|  |  | G/A-A/A | 200 (52.1%) | 43 (58.1%) | 1.37 (0.81-2.32) |  | 1.28 (0.77-2.11) |  |
|  | Recessive | G/G-G/A | 344 (89.6%) | 65 (87.8%) | 1.00 | 0.720 | 1.00 | 0.660 |
|  |  | A/A | 40 (10.4%) | 9 (12.2%) | 1.16 (0.52-2.56) |  | 1.19 (0.55-2.57) |  |
|  | Log-additive | --- | --- | --- | 1.22 (0.84-1.78) | 0.300 | 1.19 (0.82-1.71) | 0.360 |
| rs1056426 | Allele | T | 585 (76.2%) | 106 (72.6%) | 1.00 | 0.357 |  |  |
|  |  | C | 183 (23.8%) | 40 (27.4%) | 1.21 (0.81-1.80) |  |  |  |
|  | Codominant | T/T | 222 (57.8%) | 41 (56.2%) | 1.00 | 0.200 | 1.00 |  |
|  |  | C/T | 141 (36.7%) | 24 (32.9%) | 0.89 (0.50-1.56) |  | 0.92 (0.53-1.59) | 0.250 |
|  |  | C/C | 21 (5.5%) | 8 (11.0%) | 2.20 (0.88-5.51) |  | 2.06 (0.86-4.97) |  |
|  | Dominant | T/T | 222 (57.8%) | 41 (56.2%) | 1.00 | 0.870 | 1.00 | 0.790 |
|  |  | C/T-C/C | 162 (42.2%) | 32 (43.8%) | 1.05 (0.62-1.77) |  | 1.07 (0.65-1.77) |  |
|  | Recessive | T/T-C/T | 363 (94.5%) | 65 (89.0%) | 1.00 | 0.082 | 1.00 | 0.100 |
|  |  | C/C | 21 (5.5%) | 8 (11.0%) | 2.30 (0.94-5.63) |  | 2.13 (0.90-5.01) |  |
|  | Log-additive | --- | --- | --- | 1.20 (0.80-1.81) | 0.380 | 1.20 (0.81-1.79) | 0.370 |
| rs1064607 | Allele | G | 488 (63.5%) | 92 (62.2%) | 1.00 | 0.750 |  |  |
|  |  | C | 280 (36.5%) | 56 (37.8%) | 1.06(0.74-1.53) |  |  |  |
|  | Codominant | G/G | 157 (40.9%) | 27 (36.5%) | 1.00 | 0.780 | 1.00 |  |
|  |  | G/C | 174 (45.3%) | 38 (51.4%) | 1.20 (0.69-2.10) |  | 1.27 (0.74-2.18) | 0.630 |
|  |  | C/C | 53 (13.8%) | 9 (12.2%) | 0.99 (0.43-2.31) |  | 0.99 (0.44-2.23) |  |
|  | Dominant | G/G | 157 (40.9%) | 27 (36.5%) | 1.00 | 0.590 | 1.00 | 0.480 |
|  |  | G/C-C/C | 227 (59.1%) | 47 (63.5%) | 1.16 (0.68-1.97) |  | 1.20 (0.72-2.02) |  |
|  | Recessive | G/G-G/C | 331 (86.2%) | 65 (87.8%) | 1.00 | 0.780 | 1.00 | 0.700 |
|  |  | C/C | 53 (13.8%) | 9 (12.2%) | 0.89 (0.41-1.95) |  | 0.86 (0.41-1.84) |  |
|  | Log-additive | --- | --- | --- | 1.05 (0.72-1.53) | 0.810 | 1.06 (0.74-1.52) | 0.750 |
| rs3796283 | Allele | A | 450 (58.6%) | 86 (58.9%) | 1.00 | 0.944 |  |  |
|  |  | G | 318 (41.4%) | 60 (41.1%) | 0.99（0.69-1.41）） |  |  |  |
|  | Codominant | A/A | 134 (34.9%) | 24 (32.9%) | 1.00 | 0.820 | 1.00 |  |
|  |  | G/A | 182 (47.4%) | 38 (52.0%) | 1.05 (0.59-1.87) |  | 1.17 (0.67-2.04) | 0.740 |
|  |  | G/G | 68 (17.7%) | 11 (15.1%) | 0.83 (0.37-1.84) |  | 0.90 (0.42-1.95) |  |
|  | Dominant | A/A | 134 (34.9%) | 24 (32.9%) | 1.00 | 0.970 | 1.00 | 0.740 |
|  |  | G/A-G/G | 250 (65.1%) | 49 (67.1%) | 0.99 (0.57-1.72) |  | 1.09 (0.64-1.86) |  |
|  | Recessive | A/A-G/A | 316 (82.3%) | 62 (84.9%) | 1.00 | 0.540 | 1.00 | 0.580 |
|  |  | G/G | 68 (17.7%) | 11 (15.1%) | 0.80 (0.39-1.65) |  | 0.82 (0.41-1.65) |  |
|  | Log-additive | --- | --- | --- | 0.93 (0.64-1.36) | 0.720 | 0.99 (0.69-1.41) | 0.940 |
| rs2378456 | Allele | G | 419 (54.7%) | 80 (55.6%) | 1.00 | 0.850 |  |  |
|  |  | C | 347 (45.3%) | 64 (44.4%) | 0.97(0.68-1.38) |  |  |  |
|  | Codominant | G/G | 117 (30.6%) | 22 (30.6%) | 1.00 | 0.92 | 1.00 | 0.940 |
|  |  | G/C | 185 (48.3%) | 36 (50%) | 0.97 (0.53-1.77) |  | 1.03 (0.58-1.85) |  |
|  |  | C/C | 81 (21.1%) | 14 (19.4%) | 0.86 (0.40-1.82) |  | 0.92 (0.44-1.90) |  |
|  | Dominant | G/G | 117 (30.6%) | 22 (30.6%) | 1.00 | 0.820 | 1.00 | 1.000 |
|  |  | G/C-C/C | 266 (69.5%) | 50 (69.4%) | 0.93 (0.53-1.65) |  | 1.00 (0.58-1.73) |  |
|  | Recessive | G/G-G/C | 302 (78.8%) | 58 (80.6%) | 1.00 | 0.680 | 1.00 | 0.740 |
|  |  | C/C | 81 (21.1%) | 14 (19.4%) | 0.87 (0.45-1.68) |  | 0.90 (0.48-1.69) |  |
|  | Log-additive | --- | --- | --- | 0.93 (0.64-1.35) | 0.700 | 0.97 (0.68-1.38) | 0.850 |
| rs3750163 | Allele | G | 707 (92.1%) | 131 (88.5%) | 1.00 | 0.157 |  |  |
|  |  | A | 61 (7.9%) | 17 (11.5%) | 1.50(0.85-2.66) |  |  |  |
|  | Codominant | G/G | 325 (84.6%) | 59 (79.7%) | 1.00 | 0.170 | 1.00 |  |
|  |  | G/A | 57 (14.8%) | 13 (17.6%) | 1.26 (0.64-2.52) |  | 1.26 (0.65-2.44) | 0.230 |
|  |  | A/A | 2 (0.5%) | 2 (2.7%) | 7.33 (0.94-57.18) |  | 5.51 (0.76-39.88) |  |
|  | Dominant | G/G | 325 (84.6%) | 59 (79.7%) | 1.00 | 0.290 | 1.00 | 0.310 |
|  |  | G/A-A/A | 59 (15.4%) | 15 (20.3%) | 1.43 (0.74-2.76) |  | 1.40 (0.75-2.63) |  |
|  | Recessive | G/G-G/A | 382 (99.5%) | 72 (97.3%) | 1.00 | 0.078 | 1.00 | 0.120 |
|  |  | A/A | 2 (0.5%) | 2 (2.7%) | 7.04 (0.91-54.77) |  | 5.31 (0.74-38.28) |  |
|  | Log-additive | --- | --- | --- | 1.56 (0.86-2.81) | 0.150 | 1.49 (0.85-2.63) | 0.180 |
| rs9876 | Allele | G | 397 (51.5%) | 76 (51.4%) | 1.00 | 0.991 |  |  |
|  |  | A | 374 (48.5%) | 72 (48.6%) | 1.00(0.70-1.42) |  |  |  |
|  | Codominant | G/G | 108 (28.1%) | 18 (24.3%) | 1.00 | 0.60 | 1.00 |  |
|  |  | A/G | 178 (46.4%) | 40 (54.0%) | 1.26 (0.67-2.36) |  | 1.35 (0.74-2.47) | 0.480 |
|  |  | A/A | 98 (25.5%) | 16 (21.6%) | 0.93 (0.44-1.99) |  | 0.98 (0.47-2.03) |  |
|  | Dominant | G/G | 108 (28.1%) | 18 (24.3%) | 1.00 | 0.650 | 1.00 | 0.500 |
|  |  | A/G-A/A | 276 (71.9%) | 56 (75.7%) | 1.15 (0.63-2.08) |  | 1.22 (0.68-2.17) |  |
|  | Recessive | G/G-A/G | 286 (74.5%) | 58 (78.4%) | 1.00 | 0.480 | 1.00 | 0.470 |
|  |  | A/A | 98 (25.5%) | 16 (21.6%) | 0.80 (0.43-1.49) |  | 0.81 (0.44-1.47) |  |
|  | Log-additive | --- | --- | --- | 0.97 (0.68-1.40) | 0.880 | 1.00 (0.71-1.41) | 0.990 |

OR, odds ratio; 95% CI, 95% confidence interval.

*p* ≤ 0.05 indicates statistical significance.

# Additional file 1: Table S7 Relationship of clinical stage with selected 3′UTR polymorphisms in lung cancer patients

| **SNP ID** | **Model** | **Genotype** | **I-II** | **III-IV** | **Adjusted analysis** | | **Crude analysis** | |
| --- | --- | --- | --- | --- | --- | --- | --- | --- |
|  |  |  |  |  | **OR (95%CI)** | ***p*** | **OR (95%CI)** | ***p*** |
| rs2246209 | Allele | G | 109 (72.7%) | 293 (68.8%) | 1.00 | 0.373 |  |  |
|  |  | A | 41 (27.3%) | 133 (31.2%) | 1.21(0.80-1.82) |  |  |  |
|  | Codominant | G/G | 42 (56.0%) | 102 (47.9%) | 1.00 | 0.440 | 1.00 |  |
|  |  | G/A | 25 (33.3%) | 89 (41.8%) | 1.45 (0.82-2.57) |  | 1.47 (0.83-2.59) | 0.420 |
|  |  | A/A | 8 (10.7%) | 22 (10.3%) | 1.12 (0.46-2.71) |  | 1.13 (0.47-2.74) |  |
|  | Dominant | G/G | 42 (56.0%) | 102 (47.9%) | 1.00 | 0.250 | 1.00 | 0.230 |
|  |  | G/A-A/A | 33 (44.0%) | 111 (52.1%) | 1.37 (0.80-2.33) |  | 1.39 (0.82-2.35) |  |
|  | Recessive | G/G-G/A | 67 (89.3%) | 191 (89.7%) | 1.00 | 0.920 | 1.00 | 0.930 |
|  |  | A/A | 8 (10.7%) | 22 (10.3%) | 0.95 (0.41-2.25) |  | 0.96 (0.41-2.27) |  |
|  | Log-additive | --- | --- | --- | 1.18 (0.79-1.77) | 0.410 | 1.19 (0.80-1.79) | 0.380 |
| rs1056426 | Allele | T | 119 (79.3%) | 324 (76.8%) | 1.00 | 0.520 |  |  |
|  |  | C | 31 (20.7%) | 98 (23.2%) | 1.16(0.74-1.83) |  |  |  |
|  | Codominant | T/T | 48 (64.0%) | 128 (60.7%) | 1.00 | 0.830 | 1.00 |  |
|  |  | C/T | 23 (30.7%) | 68 (32.2%) | 1.11 (0.62-1.98) |  | 1.11 (0.62-1.98) | 0.810 |
|  |  | C/C | 4 (5.3%) | 15 (7.1%) | 1.37 (0.43-4.36) |  | 1.41 (0.44-4.45) |  |
|  | Dominant | T/T | 48 (64.0%) | 128 (60.7%) | 1.00 | 0.620 | 1.00 | 0.610 |
|  |  | C/T-C/C | 27 (36.0%) | 83 (39.3%) | 1.15 (0.66-1.98) |  | 1.15 (0.67-1.99) |  |
|  | Recessive | T/T-C/T | 71 (94.7%) | 196 (92.9%) | 1.00 | 0.620 | 1.00 | 0.590 |
|  |  | C/C | 4 (5.3%) | 15 (7.1%) | 1.33 (0.43-4.14) |  | 1.36 (0.44-4.23) |  |
|  | Log-additive | --- | --- | --- | 1.14 (0.74-1.77) | 0.550 | 1.15 (0.74-1.78) | 0.530 |
| rs1064607 | Allele | G | 96 (64.0%) | 246 (58.0%) | 1.00 | 0.200 |  |  |
|  |  | C | 54 (36.0%) | 178 (42.0%) | 1.29(0.88-1.89) |  |  |  |
|  | Codominant | G/G | 32 (42.7%) | 68 (32.1%) | 1.00 | 0.240 | 1.00 |  |
|  |  | G/C | 32 (42.7%) | 110 (51.9%) | 1.64 (0.92-2.94) |  | 1.62 (0.91-2.88) | 0.250 |
|  |  | C/C | 11 (14.7%) | 34 (16.0%) | 1.44 (0.64-3.20) |  | 1.45 (0.65-3.23) |  |
|  | Dominant | G/G | 32 (42.7%) | 68 (32.1%) | 1.00 | 0.099 | 1.00 | 0.100 |
|  |  | G/C-C/C | 43 (57.3%) | 144 (67.9%) | 1.59 (0.92-2.74) |  | 1.58 (0.92-2.71) |  |
|  | Recessive | G/G-G/C | 64 (85.3%) | 178 (84.0%) | 1.00 | 0.810 | 1.00 | 0.780 |
|  |  | C/C | 11 (14.7%) | 34 (16.0%) | 1.09 (0.52-2.29) |  | 1.11 (0.53-2.32) |  |
|  | Log-additive | --- | --- | --- | 1.29 (0.87-1.92) | 0.200 | 1.00 | 0.170 |
| rs3796283 | Allele | A | 89 (59.3%) | 229 (53.8%) | 1.00 | 0.237 |  |  |
|  |  | G | 61 (40.7%) | 197 (46.2%) | 1.26(0.86-1.83) |  |  |  |
|  | Codominant | A/A | 27 (36.0%) | 57 (26.8%) | 1.00 | 0.330 | 1.00 |  |
|  |  | G/A | 35 (46.7%) | 115 (54.0%) | 1.56 (0.86-2.85) |  | 1.56 (0.86-2.82) | 0.330 |
|  |  | G/G | 13 (17.3%) | 41 (19.2%) | 1.50 (0.69-3.27) |  | 1.49 (0.69-3.24) |  |
|  | Dominant | A/A | 27 (36.0%) | 57 (26.8%) | 1.00 | 0.140 | 1.00 | 0.130 |
|  |  | G/A-G/G | 48 (64.0%) | 156 (73.2%) | 1.55 (0.88-2.73) |  | 1.54 (0.88-2.70) |  |
|  | Recessive | A/A-G/A | 62 (82.7%) | 172 (80.8%) | 1.00 | 0.700 | 1.00 | 0.710 |
|  |  | G/G | 13 (17.3%) | 41 (19.2%) | 1.14 (0.57-2.28) |  | 1.14 (0.57-2.26) |  |
|  | Log-additive | --- | --- | --- | 1.27 (0.86-1.88) | 0.220 | 1.27 (0.86-1.88) | 0.220 |
| rs2378456 | Allele | G | 80 (55.6%) | 218 (51.7%) | 1.00 | 0.419 |  |  |
|  |  | C | 64 (44.4%) | 204 (48.3%) | 1.17(0.80-1.71) |  |  |  |
|  | Codominant | G/G | 23 (31.9%) | 55 (26.1%) | 1.00 | 0.630 | 1.00 | 0.630 |
|  |  | G/C | 34 (47.2%) | 108 (51.2%) | 1.34 (0.71-2.52) |  | 1.33 (0.71-2.47) |  |
|  |  | C/C | 15 (20.8%) | 48 (22.8%) | 1.36 (0.63-2.91) |  | 1.34 (0.63-2.85) |  |
|  | Dominant | G/G | 23 (31.9%) | 55 (26.1%) | 1.00 | 0.330 | 1.00 | 0.340 |
|  |  | G/C-C/C | 49 (68.1%) | 156 (73.9%) | 1.34 (0.74-2.43) |  | 1.33 (0.74-2.39) |  |
|  | Recessive | G/G-G/C | 57 (79.2%) | 163 (77.2%) | 1.00 | 0.710 | 1.00 | 0.730 |
|  |  | C/C | 15 (20.8%) | 48 (22.8%) | 1.13 (0.59-2.18) |  | 1.12 (0.58-2.15) |  |
|  | Log-additive | --- | --- | --- | 1.18 (0.80-1.73) | 0.410 | 1.17 (0.80-1.72) | 0.420 |
| rs3750163 | Allele | G | 138 (92.0%) | 388 (91.1%) | 1.00 | 0.731 |  |  |
|  |  | A | 12 (8.0%) | 38 (8.9%) | 1.13(0.57-2.22) |  |  |  |
|  | Codominant | G/G | 65 (86.7%) | 177 (83.1%) | 1.00 | 0.250 | 1.00 |  |
|  |  | G/A | 8 (10.7%) | 34 (16.0%) | 1.61 (0.71-3.68) |  | 1.56 (0.69-3.55) | 0.320 |
|  |  | A/A | 2 (2.7%) | 2 (0.9%) | 0.31 (0.04-2.31) |  | 0.37 (0.05-2.66) |  |
|  | Dominant | G/G | 65 (86.7%) | 177 (83.1%) | 1.00 | 0.440 | 1.00 | 0.460 |
|  |  | G/A-A/A | 10 (13.3%) | 36 (16.9%) | 1.34 (0.63-2.85) |  | 1.32 (0.62-2.82) |  |
|  | Recessive | G/G-G/A | 73 (97.3%) | 211 (99.1%) | 1.00 | 0.240 | 1.00 | 0.300 |
|  |  | A/A | 2 (2.7%) | 2 (0.9%) | 0.29 (0.04-2.19) |  | 0.35 (0.05-2.50) |  |
|  | Log-additive | --- | --- | --- | 1.12 (0.58-2.15) | 0.740 | 1.12 (0.58-2.15) | 0.740 |
| rs9876 | Allele | G | 75 (50.0%) | 231 (54.5%) | 1.00 | 0.344 |  |  |
|  |  | A | 75 (50.0%) | 193 (45.5%) | 0.84(0.58-1.21) |  |  |  |
|  | Codominant | G/G | 20 (26.7%) | 60 (28.3%) | 1.00 | 0.410 | 1.00 |  |
|  |  | A/G | 35 (46.7%) | 111 (52.4%) | 1.05 (0.56-1.98) |  | 1.06 (0.56-1.99) | 0.420 |
|  |  | A/A | 20 (26.7%) | 41 (19.3%) | 0.68 (0.32-1.42) |  | 0.68 (0.33-1.43) |  |
|  | Dominant | G/G | 20 (26.7%) | 60 (28.3%) | 1.00 | 0.770 | 1.00 | 0.790 |
|  |  | A/G-A/A | 55 (73.3%) | 152 (71.7%) | 0.91 (0.51-1.66) |  | 0.92 (0.51-1.67) |  |
|  | Recessive | G/G-A/G | 55 (73.3%) | 171 (80.7%) | 1.00 | 0.190 | 1.00 | 0.190 |
|  |  | A/A | 20 (26.7%) | 41 (19.3%) | 0.66 (0.35-1.22) |  | 0.66 (0.36-1.22) |  |
|  | Log-additive | --- | --- | --- | 0.83 (0.57-1.21) | 0.330 | 0.83 (0.57-1.21) | 0.340 |

OR, odds ratio; 95% CI, 95% confidence interval.

*p* ≤ 0.05 indicates statistical significance.

# Additional file 1: Table S8 Relationship of lymphatic metastatic status with 3′UTR polymorphisms in lung cancer patients

| **SNP ID** | **Model** | **Genotype** | **Non-Metastasis** | **Metastasis** | **Adjusted analysis** | | **Crude analysis** | |
| --- | --- | --- | --- | --- | --- | --- | --- | --- |
|  |  |  |  |  | **OR (95%CI)** | ***p*** | **OR (95%CI)** | ***p*** |
| rs2246209 | Allele | G | 175 (68.9%) | 275 (70.5%) | 1.00 | 0.662 |  |  |
|  |  | A | 79 (31.1%) | 115 (29.5%) | 0.93(0.66-1.31) |  |  |  |
|  | Codominant | G/G | 63 (49.6%) | 97 (49.7%) | 1.00 | 0.630 | 1.00 |  |
|  |  | G/A | 49 (38.6%) | 81 (41.5%) | 1.08 (0.67-1.75) |  | 1.07 (0.67-1.73) | 0.640 |
|  |  | A/A | 15 (11.8%) | 17 (8.7%) | 0.74 (0.34-1.59) |  | 0.74 (0.34-1.58) |  |
|  | Dominant | G/G | 63 (49.6%) | 97 (49.7%) | 1.00 | 0.990 | 1.00 | 0.980 |
|  |  | G/A-A/A | 64 (50.4%) | 98 (50.3%) | 1.00 (0.64-1.57) |  | 0.99 (0.64-1.56) |  |
|  | Recessive | G/G-G/A | 112 (88.2%) | 178 (91.3%) | 1.00 | 0.370 | 1.00 | 0.370 |
|  |  | A/A | 15 (11.8%) | 17 (8.7%) | 0.71 (0.34-1.49) |  | 0.71 (0.34-1.48) |  |
|  | Log-additive | --- | --- | --- | 0.93 (0.66-1.31) | 0.690 | 0.93 (0.66-1.30) | 0.670 |
| rs1056426 | Allele | T | 192 (76.2%) | 304 (78.4%) | 1.00 | 0.523 |  |  |
|  |  | C | 60 (23.8%) | 84 (21.6%) | 0.88(0.61-1.29) |  |  |  |
|  | Codominant | T/T | 76 (60.3%) | 121 (62.4%) | 1.00 | 0.720 | 1.00 |  |
|  |  | C/T | 40 (31.8%) | 62 (32.0%) | 0.98 (0.60-1.61) |  | 0.97 (0.60-1.59) | 0.730 |
|  |  | C/C | 10 (7.9%) | 11 (5.7%) | 0.69 (0.28-1.70) |  | 0.69 (0.28-1.70) |  |
|  | Dominant | T/T | 76 (60.3%) | 121 (62.4%) | 1.00 | 0.740 | 1.00 | 0.710 |
|  |  | C/T-C/C | 50 (39.7%) | 73 (37.6%) | 0.92 (0.58-1.47) |  | 0.92 (0.58-1.45) |  |
|  | Recessive | T/T-C/T | 116 (92.1%) | 183 (94.3%) | 1.00 | 0.420 | 1.00 | 0.430 |
|  |  | C/C | 10 (7.9%) | 11 (5.7%) | 0.69 (0.28-1.68) |  | 0.70 (0.29-1.69) |  |
|  | Log-additive | --- | --- | --- | 0.90 (0.62-1.29) | 0.550 | 0.89 (0.62-1.28) | 0.540 |
| rs1064607 | Allele | G | 158 (62.2%) | 224 (57.7%) | 1.00 | 0.259 |  |  |
|  |  | C | 96 (37.8%) | 164 (42.3%) | 1.20(0.87-1.67) |  |  |  |
|  | Codominant | G/G | 50 (39.4%) | 61 (31.4%) | 1.00 | 0.290 | 1.00 |  |
|  |  | G/C | 58 (45.7%) | 102 (52.6%) | 1.49 (0.91-2.45) |  | 1.44 (0.88-2.36) | 0.340 |
|  |  | C/C | 19 (15.0%) | 31 (16.0%) | 1.34 (0.67-2.65) |  | 1.34 (0.68-2.65) |  |
|  | Dominant | G/G | 50 (39.4%) | 61 (31.4%) | 1.00 | 0.120 | 1.00 | 0.150 |
|  |  | G/C-C/C | 77 (60.6%) | 133 (68.6%) | 1.45 (0.91-2.33) |  | 1.42 (0.89-2.26) |  |
|  | Recessive | G/G-G/C | 108 (85.0%) | 163 (84.0%) | 1.00 | 0.850 | 1.00 | 0.810 |
|  |  | C/C | 19 (15.0%) | 31 (16.0%) | 1.06 (0.57-1.98) |  | 1.08 (0.58-2.01) |  |
|  | Log-additive | --- | --- | --- | 1.22 (0.88-1.70) | 0.240 | 1.21 (0.87-1.69) | 0.250 |
| rs3796283 | Allele | A | 142 (55.9%) | 213 (54.9%) | 1.00 | 0.802 |  |  |
|  |  | G | 112 (44.1%) | 175 (45.1%) | 1.04(0.76-1.43) |  |  |  |
|  | Codominant | A/A | 39 (30.7%) | 55 (28.4%) | 1.00 | 0.830 | 1.00 |  |
|  |  | G/A | 64 (50.4%) | 103 (53.1%) | 1.18 (0.70-1.98) |  | 1.14 (0.68-1.91) | 0.880 |
|  |  | G/G | 24 (18.9%) | 36 (18.6%) | 1.08 (0.56-2.10) |  | 1.06 (0.55-2.06) |  |
|  | Dominant | A/A | 39 (30.7%) | 55 (28.4%) | 1.00 | 0.580 | 1.00 | 0.650 |
|  |  | G/A-G/G | 88 (69.3%) | 139 (71.7%) | 1.15 (0.70-1.88) |  | 1.12 (0.69-1.83) |  |
|  | Recessive | A/A-G/A | 103 (81.1%) | 158 (81.4%) | 1.00 | 0.930 | 1.00 | 0.940 |
|  |  | G/G | 24 (18.9%) | 36 (18.6%) | 0.98 (0.55-1.73) |  | 0.98 (0.55-1.73) |  |
|  | Log-additive | --- | --- | --- | 1.05 (0.76-1.47) | 0.750 | 1.04 (0.75-1.45) | 0.800 |
| rs2378456 | Allele | G | 132 (53.2%) | 201 (52.3%) | 1.00 | 0.828 |  |  |
|  |  | C | 116 (46.8%) | 183 (47.7%) | 1.04(0.75-1.43) |  |  |  |
|  | Codominant | G/G | 36 (29.0%) | 52 (27.1%) | 1.00 | 0.860 | 1.00 | 0.920 |
|  |  | G/C | 60 (48.4%) | 97 (50.5%) | 1.16 (0.68-2.00) |  | 1.12 (0.66-1.91) |  |
|  |  | C/C | 28 (22.6%) | 43 (22.4%) | 1.09 (0.57-2.08) |  | 1.06 (0.56-2.01) |  |
|  | Dominant | G/G | 36 (29.0%) | 52 (27.1%) | 1.00 | 0.620 | 1.00 | 0.710 |
|  |  | G/C-C/C | 88 (71.0%) | 140 (72.9%) | 1.14 (0.68-1.90) |  | 1.10 (0.67-1.82) |  |
|  | Recessive | G/G-G/C | 96 (77.4%) | 149 (77.6%) | 1.00 | 0.980 | 1.00 | 0.970 |
|  |  | C/C | 28 (22.6%) | 43 (22.4%) | 0.99 (0.58-1.71) |  | 0.99 (0.58-1.70) |  |
|  | Log-additive | --- | --- | --- | 1.05 (0.76-1.45) | 0.760 | 1.04 (0.75-1.43) | 0.830 |
| rs3750163 | Allele | G | 229 (90.2%) | 359 (92.1%) | 1.00 | 0.405 |  |  |
|  |  | A | 25 (9.8%) | 31 (7.9%) | 0.79(0.46-1.37) |  |  |  |
|  | Codominant | G/G | 105 (82.7%) | 165 (84.6%) | 1.00 | 0.340 | 1.00 |  |
|  |  | G/A | 19 (15%) | 29 (14.9%) | 0.99 (0.53-1.86) |  | 0.97 (0.52-1.82) | 0.350 |
|  |  | A/A | 3 (2.4%) | 1 (0.5%) | 0.20 (0.02-2.04) |  | 0.21 (0.02-2.07) |  |
|  | Dominant | G/G | 105 (82.7%) | 165 (84.6%) | 1.00 | 0.680 | 1.00 | 0.650 |
|  |  | G/A-A/A | 22 (17.3%) | 30 (15.4%) | 0.88 (0.48-1.61) |  | 0.87 (0.48-1.58) |  |
|  | Recessive | G/G-G/A | 124 (97.6%) | 194 (99.5%) | 1.00 | 0.140 | 1.00 | 0.150 |
|  |  | A/A | 3 (2.4%) | 1 (0.5%) | 0.20 (0.02-2.04) |  | 0.21 (0.02-2.07) |  |
|  | Log-additive | --- | --- | --- | 0.81 (0.47-1.39) | 0.440 | 0.80 (0.47-1.37) | 0.420 |

OR = odds ratio; 95% CI = 95% confidence interval.

*p* ≤ 0.05 indicates statistical significance.

# Additional file 1: Table S9 Haplotype frequencies and their associations with lung cancer risk

| **Haplotype** | **Frequency** | **Crude analysis** | | **Adjusted by age and gender** | |
| --- | --- | --- | --- | --- | --- |
|  |  | **OR (95% CI)** | ***p*** | **OR (95% CI)** | ***p*** |
| GAG | 0.524 | 1.00 |  | 1.00 |  |
| CGC | 0.370 | 1.15 (0.92-1.44) | 0.220 | 1.13 (0.89-1.44) | 0.310 |
| GGC | 0.051 | 1.01 (0.61-1.66) | 0.970 | 0.90 (0.52-1.54) | 0.690 |
| GAC | 0.041 | 1.03 (0.59-1.80) | 0.910 | 0.98 (0.54-1.78) | 0.960 |
| rare | 0.014 | **4.49 (1.48-13.61)** | **0.008** | **6.17 (1.76-21.68)** | **0.004** |

The block comprises the three closely linked SNPs rs1064607, rs3796283and rs2378456. OR, odds ratio; 95% CI, 95% confidence interval.

*p* < 0.05 indicates statistical significance.
